# COVID-19 case doubling time associated with non-pharmaceutical interventions and vaccination: A global experience

**DOI:** 10.7189/jogh.11.05021

**Published:** 2021-09-04

**Authors:** Li-Lin Liang, Chien-Tse Kao, Hsiu J Ho, Chun-Ying Wu

**Affiliations:** 1 Department of Business Management, National Sun Yat-sen University, Kaohsiung, Taiwan; 2 Research Center for Epidemic Prevention, National Yang Ming Chiao Tung University, Taipei, Taiwan; 3 Austin Hospital, Austin Health, Melbourne, Australia; 4 Institute of Biomedical Informatics, National Yang Ming Chiao Tung University, Taipei, Taiwan; 5 Division of Translational Research, Taipei Veterans General Hospital, Taipei, Taiwan; 6 Department of Public Health, China Medical University, Taichung, Taiwan

## Abstract

**Background:**

Evidence has revealed that nonpharmaceutical interventions (NPIs) were effective in attenuating the spread of COVID-19. However, policymakers have encountered difficulty in identifying the most effective policies under different circumstances. This study investigated the relative effectiveness of different NPIs and vaccination in prolonging COVID-19 case doubling time (DT).

**Methods:**

The study sample consisted of observations from 137 countries during 1 January 2020 to 13 June 2021. DT was calculated on a daily basis per country. Data were retrieved from the Oxford COVID-19 Government Response Tracker, World Development Indicators, and Worldwide Governance Indicators. To capture policy intervention dynamics, we combined a random-effect growth-curve model with nonstandard interrupted time series analysis. We also evaluated the association of policy measures with DT for different outbreak stages and levels of government effectiveness.

**Results:**

Vaccine rollouts, workplace closures, and school closures were relatively effective. For each day that these measures were implemented, the DT increased by 1.96% (95% confidence interval (CI) = 0.63 to 3.29; *P* = 0.004), 1.41% (95% CI = 0.88 to 1.95%; *P* < 0.001) and 1.38% (95% CI = 0.95 to 1.81%; *P* < 0.001), respectively. Workplace and school closures were positively associated with DT at all stages; however, the associations weakened in later stages, where vaccine rollouts appeared to be most effective in prolonging DT (95% CI = 1.51% to 3.04%; *P* < 0.05). For countries with a high level of government effectiveness, most of the containment measures evaluated were effective; vaccine rollouts had the greatest effect size. For countries with medium or low levels of government effectiveness, only the closure of workplaces was consistently associated with prolonged DT.

**Conclusions:**

The effectiveness of vaccine rollouts outweighed that of NPIs, especially in the later outbreak stages. However, vaccination was not associated with prolonged case DT in countries with lower levels of government effectiveness, probably due to low vaccine coverage. Among the NPIs examined, workplace closures were highly effective across all outbreak stages and levels of government effectiveness. Our findings suggest that mass vaccination is critical to reducing SARS-CoV-2 transmission, especially in countries where NPIs are less effective.

Since the outbreak of coronavirus disease 2019 (COVID-19) in early 2020, policymakers have been confronted with the task of balancing the tradeoff between saving lives and saving economies. Containment measures, including travel restrictions and physical distancing, have been imposed to curb COVID-19 infection rates [1-3]. However, these measures have threatened employment and other economic activity. Efforts are growing to end the pandemic through mass vaccination. As of 27 June 2021, 23.1% of the world population has received at least one dose of a COVID-19 vaccine; however, the coverage is far from that required for global herd immunity [4]. Before effective drugs and vaccines become accessible to most populations, nonpharmaceutical interventions (NPIs) will remain key strategies for flattening the pandemic curve. Against this backdrop, understanding the effectiveness of policy measures under different circumstances is imperative.

This study investigated the effectiveness of a comprehensive set of NPIs and vaccine rollouts in prolonging the COVID-19 case doubling time (DT). Literature examining COVID-19 NPIs has disproportionately focused on the first half of 2020; Hsiang et al. discovered that anti-contagion policies implemented in six countries slowed the spread of COVID-19 from its initial outbreak until early April [5]. In an evaluation of 11 European countries, Flaxman et al. confirmed that containment interventions reduced COVID-19 transmission from February until early May [6]. Islam et al. examined the effects of containment measures in 149 countries from the first outbreak until the end of May [7]. Brauner et al. estimated the effectiveness of NPIs for 34 European and 7 non-European countries between January and May 2020 [8], and Haug et al. assessed the effectiveness of worldwide NPIs implemented in March-April 2020 [9]. Other studies have examined the effects of NPIs during spring 2020 [10-13]. More recently, scholars have reported the combined effects of NPIs and vaccine rollouts based on simulations of epidemiological models for the United Kingdom [14], the United States [15], Italy [16], and China [17]. Studies have demonstrated that mass vaccination significantly reduced SARS-CoV-2 infection rates and hospitalisations in Israel [18] and Scotland [19].

After June 2020, COVID-19 spread exponentially worldwide, and the effectiveness of NPIs was influenced by governance and local contexts [9]. Building on existing NPI research, our investigation extended the time horizon and range of policy measures, using daily observations from 137 countries over 18 months from January 2020 to June 2021. The scale and intensity of health and containment measures changed, and vaccination programmes were introduced in many countries. How policy effects changed with governance quality is unclear. This study aims to understand (a) the relative effectiveness of NPIs and vaccine rollouts, and (b) changes in policy effects over time and across countries. Our previous work demonstrated that government effectiveness is negatively associated with COVID-19 mortality [20]. In the present study, we further explored policy effects across different outbreak stages and levels of government effectiveness. Crucially, the present research complements the aforementioned simulation studies by using real-world data across countries. Our findings may serve as a basis for future analyses that policymakers can reference to make optimal policy decisions.

## METHODS

We used a random-effect growth-curve model combined with nonstandard interrupted time series analysis to capture the dynamics of policy interventions. The log of DT was specified as a quadratic function of time and policy variables, government effectiveness, intrinsic country characteristics, as well as country-specific random intercepts and random coefficients of time. Data sources and econometric methods used are described as follows.

### Data collection and study sample

Data were collected on 19 June 2021 from three open-access databases: the Oxford COVID-19 Government Response Tracker (OxCGRT) [21], World Development Indicators [22], and Worldwide Governance Indicators [23]. Data for more recent dates were incomplete; thus the observation period was from 1 January 2020 to 13 June 2021. We included countries with data in all three databases and excluded countries or regions that met the following criteria: (a) fewer than 500 confirmed cases as of 13 June 2021; (b) missing data in the OxCGRT database for more than 90 days; (c) fewer than 1 million populations, and (d) fewer than 40 observations of the DT variable (see next section). The final sample was a panel data set of 137 countries, or 42 102 country-days, since the first reported case in each country. The final date of the study period differed for individual countries due to calculation of DT. A flowchart of sample selection process is provided in Appendix S1 in the **Online Supplementary Document**. Appendix S2 in the **Online Supplementary Document** provides a list of sample countries.

### Calculation of the case doubling time

The COVID-19 case DT was defined as the number of days required for the accumulated case number to double. We focused on DT rather than case number because DT could capture delayed effects of policy interventions.

The DT was calculated on a daily basis for individual countries until the most recent date at which the result of doubling the case number exceeded the case number on 13 June 2021. Take the United States as an example. The first date for the DT variable was 22 January 2020, when the first case was reported. On 15 December 2020, the case number was 16 837 160 and its doubled value (33,674,320) exceeded the case number on 13 June 2021 (33,461,982). Thus, the United States had 328 observations or days (22 January to 14 December 2020) in our sample. To calculate the DT variable, we retrieved data regarding daily case number from the OxCGRT database. In our sample, the number of observations per country ranged from 46 to 488 (see Appendix S2 in **the** **Online Supplementary Document**). Because DT was skewed, we log-transformed the variable for regression analysis.

### Data on COVID-19 policy measures

Data on COVID-19 policy measures were retrieved from the OxCGRT, a comprehensive database established by Blavatnik School of Government, University of Oxford [24]. The database provides a systematic means of comparing government responses across countries over time. It has 18 policy indicators based on publicly available information and categorises these indicators into three domains: containment and closure, economic measures, and health measures [25]. We used six indicators in the containment domain, namely, school closures, workplace closures, cancellation of public events, restrictions on gathering size, requirements to stay-at-home, and restrictions on international travel [25]. In addition, we used five indicators related to health measures: public information campaigns, testing policy, contact tracing, face covering, and vaccine rollouts [25]. The OxCGRT has recorded indicators daily for 186 countries since the 1 January 2020.

All indicators were given values on an ordinal scale of 0-2, 0-3, 0-4, or 0-5. A value of 0 refers to no policy, and a higher positive value indicates a higher degree of policy stringency or intensity. For example, for school closures, 0 refers to ‘no measures’, 1 refers to ‘recommend closure’, 2 refers to ‘required closure for some levels or categories’, and 3 refers to ‘required closure at all levels’. We focused on interventions that were actually enforced; thus for most indicators, degree of 0 and 1 were treated as the reference group, ie, no measures. In addition, we combined the highest two degrees for stay-at-home requirements, testing policies, and face covering. For vaccine rollouts, we combined degree of 1 − 5 into a single category because the number of country-days for individual degrees was relatively small.

Appendix S3 in the **Online Supplementary Document** provides the definition of policy measures based on the OxCGRT indicators. Appendix S4 in the **Online Supplementary Document** summarises frequency and average duration of individual policy measures. In addition, we included the timeliness of interventions, defined as the number of weeks that elapsed between the date of the first confirmed death and the date of implementation of the first containment measure. The OxCGRT contains records of the daily number of deaths.

### Government effectiveness data

Government effectiveness data were retrieved from the Worldwide Governance Indicators website, which has reported governance indicators for 214 countries and territories annually since 1996 for six domains: government effectiveness, regulatory quality, control of corruption, rule of law, voice and accountability, and political stability and absence of violence [26]. Worldwide Governance Indicators were developed by scholars from the Natural Resource Governance Institute, the Brookings Institution, and the World Bank. To create these indicators, researchers rescaled and combined data from different sources, including surveys of households and firms and expert assessments, using an unobserved components model [26].

We used 2019 data for government effectiveness scores, which measure ‘the quality of public services, the quality of the civil service and the degree of its independence from political pressures, the quality of policy formulation and implementation, and the credibility of the government’s commitment to such policies’ [26] (in page 4 of the citing paper). Scores range from −2.5 to 2.5, where a higher score indicates greater effectiveness. We multiplied scores by 10 for the convenience of interpretation of the regression coefficient. In 2019, the top three countries with the highest government effectiveness scores were Singapore, Switzerland, and Denmark (Appendix S2 in the **Online Supplementary Document**).

### Country characteristics data

The variables for country characteristics used in this study were as follows: total population (log value); the percentage of the population aged 65 years or older (%); population density, measured as the number of people per square kilometre of land (in hundreds); universal health coverage service coverage index (from 0 to 100); gross domestic product per capita, adjusted according to purchasing power parity (in current international dollars); the percentage of deaths among people of all ages caused by communicable diseases or maternal, prenatal, or nutrition conditions (%). All these variables were retrieved from the World Development Indicators database [22]. The most recent available year for country characteristics data up to year 2019 was used. In addition, to capture factors that vary according to geographical location, including temperature and culture differences, we included five indicators for six continents: Europe, Africa, Asia, North America, South America, and Oceania.

### Growth-curve model and interrupted time series analysis

Primary challenges of estimation are that multiple interventions were concurrently in place or introduced with different intensities at different points in time. Policies tended to be revised or lifted after being introduced. Furthermore, finding a country that had not adopted any containment policies to serve as the comparison group was difficult.

To address these problems, we combined a growth-curve model with interrupted time series analysis (ITSA). Growth-curve models, known as latent-trajectory models, are a multilevel approach to analysing longitudinal data [27]. ITSA is a quasi-experimental research design with a potentially high degree of internal validity [28]. We applied the growth-curve model to predict the trajectory of the case DT in the absence of interventions, and ITSA to estimate policy effects.

The empirical model is specified as:


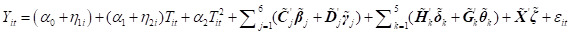
 (1), 

where *Y_it_* refers to the log of the case DT for country *i* at date *t*; *T_it_* is the number of days since the first reported case, and 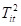 is the squared term. The polynomial function of time permits a nonlinear trend in DT. Their coefficients (*α_1_*, *α_2_*) represent the common trend of the DT in the absence of any policy intervention. To enable the evaluation of different trajectories in different countries, we included a country-specific random intercept *η_1i_* and a random coefficient of time or random slope *η_2i_*. Random intercepts and slopes capture differences in baseline DT and deviations from common trends across countries, respectively. An advantage of introducing random effects is that unobservable country-specific factors, such as the systematic underreporting of case numbers, can be accounted for. *ε_it_* is the typical idiosyncratic error.

In line with ITSA, we used each country’s pre-intervention period as the counterfactual and attributed changes in the trend during any intervention period to policy effects. In standard ITSA, policy variables often take the form as binary variables to indicate post-intervention periods. Here we extended the standard ITSA by creating a unique set of variables to capture the dynamics of policy interventions. These variables measure the number of days since the first day of the most recent implementation of specific policy with a specific intensity recorded in the OxCGRT database, as denoted by vector 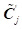 for containment measure *j* and 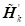 for health measure *k*. For instance, 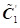 consists of the number of days since the first day of the most recent implementation of partial and full school closures, respectively, in country *i* at date *t*. When an intervention was not in place at date *t*, the corresponding policy variable was equal to 0. This method enabled us to capture policy dynamics by focusing on multiple independent intervention periods while accounting for policy cancellations. Vectors 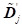 and 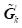 denote the vector of squared terms of the corresponding policy variables. Finally, vector 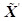 contains the variables of government effectiveness score, country characteristics, and the timeliness of interventions.

The primary interests of estimation were the coefficients of policy variables. The percentage change in case DT for each day of implementation, ie, the effect size of the corresponding policy measure (when disregarding the policy squared terms) was (100 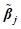)% and (100 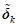)%. We estimated Equation (1) by using the maximum likelihood method. The likelihood-ratio test rejects the null hypothesis that the random coefficients are all zero (*P* < 0.001), supporting the inclusion of random slopes. For empirical estimation, we did not assume any specific structure of the variance-covariance matrix of the random intercept and random slope and allowed for intragroup (within-country) correlations when estimating standard errors.

Descriptive statistics of the model variables are presented in Appendix S5 in the **Online Supplementary Document**. In addition, Appendix S6 in the **Online Supplementary Document** presents the number of countries that implemented each of the 11 policy measures according to calendar date. All estimations were performed using Stata 16 software (Stata Corp Inc., College Station, Texas, USA).

### Subgroup analysis of heterogeneous policy effects

The policy effect may vary across different disease outbreak stages. Therefore, we performed subsample regressions for periods before a country reached the following thresholds: <5,000, <20,000, <80,000, <320 000 and <1 280 000 cases. Additionally, to explore whether the effect of a policy measure varied with government effectiveness, we categorised countries into high, medium, and low government effectiveness groups with an equal size (N = 46, 45, 46) and performed subsample regressions.

## RESULTS

### Associations of policy measures with case DT based on the full sample

**Figure 1:**
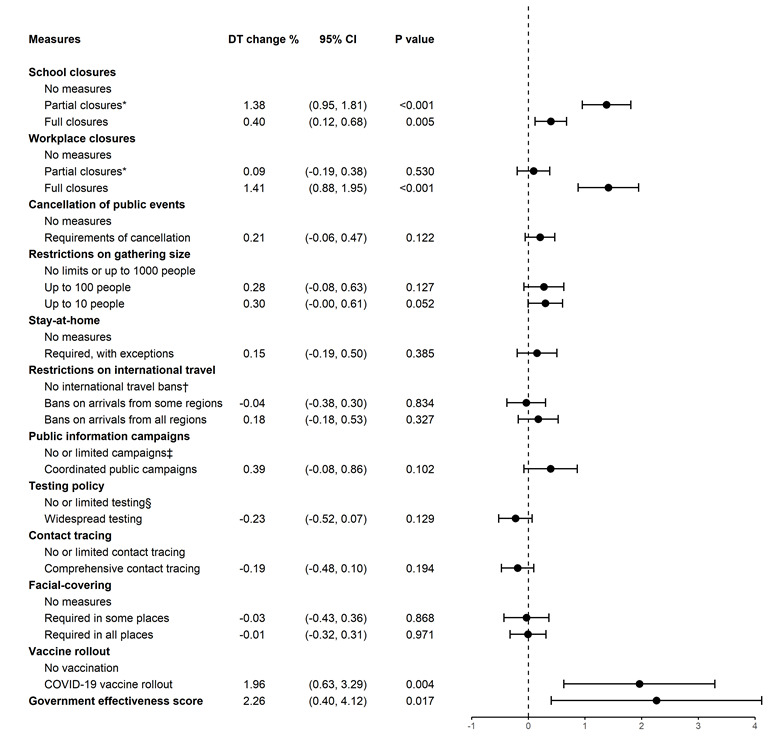
Associations of NPIs and vaccine rollouts with case doubling time (DT). The first column presents policy variables constructed from the Oxford COVID-19 Government Response Tracker. The second column is the estimated policy effect, ie, the percentage change of DT for each day of intervention. The most effective measure was vaccine rollouts, which increased DT by 1.96% for each day of intervention. The length of bar on the right represents the 95% confidence interval; the dot in the middle represents the estimated policy effect. *Partial closures means closures were required for some levels, sectors or regions. †This category includes policies of testing or quarantine on arrival. ‡Limited campaigns means only public officials urging caution about COVID-19. §Limited testing means tests were available only for certain groups of people, eg, inpatients.

The association of different policy measures with COVID-19 case DT is illustrated in **Figure 1**. Because the coefficient estimates for the policy quadratic terms in Equation (1) were trivial, we did not present them. The most effective measures were vaccine rollouts, full closure of (nonessential) workplaces, and partial school closures. For each day that these measures were in place, the DT increased by 1.96% (95% CI = 0.63 to 3.29; *P* = 0.004), 1.41% (95% CI = 0.88 to 1.95%; *P* < 0.001) and 1.38% (95% CI = 0.95 to 1.81%; *P* < 0.001), respectively. Moreover, countries that had a lower level of government effectiveness, a larger population size, a higher population density, and higher per-capita GDP appeared to have shorter case DT. Compared with European countries, Oceania countries, including Australia and New Zealand had longer DT. Full results are presented in Appendix S7 in the **Online Supplementary Document**.

### Associations between policy measures and case DT vary with COVID-19 outbreak stage

**Figure 2:**
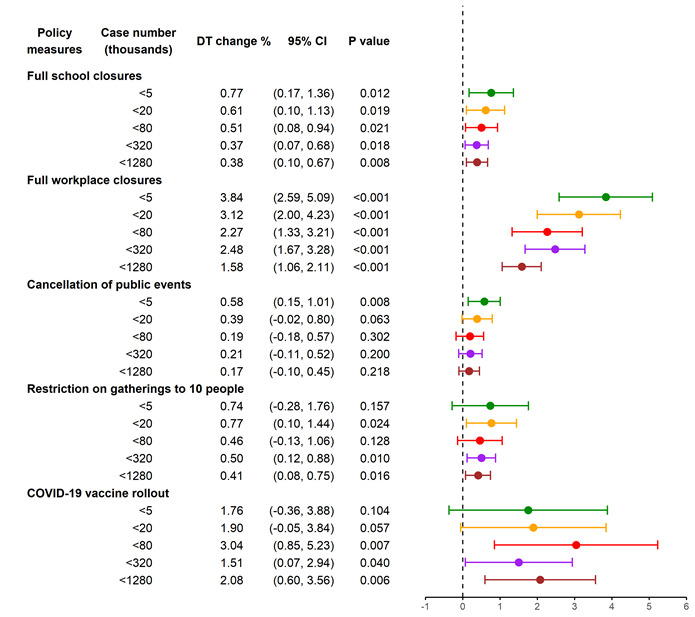
Associations of NPIs and vaccine rollouts with case doubling time (DT), by outbreak stage. The first column presents policy variables constructed from the Oxford COVID-19 Government Response Tracker. The second column presents the threshold used in terms of accumulated case number to define the outbreak stage. The third column is the estimated policy effect, ie, the percentage change in DT for each day of intervention. The length of bar on the right represents the 95% confidence interval; the dot in the middle represents the estimated policy effect. This figure illustrates only policies with effect sizes consistently decreasing with outbreak stages. The sample is a panel comprising of 137 countries.

The subgroup regressions indicate that for most policy measures, the associations with prolonged DT were stronger in earlier outbreak stages, except vaccine rollouts. **Figure 2** illustrates containment measures that had an effect size that consistently decreased across outbreak stages. For example, when the COVID-19 cases were fewer than 5000, full closure of schools and workplaces per day prolonged the DT by 0.77% (95% CI = 0.17 to 1.36%; *P* = 0.012) and 3.84% (95% CI = 2.59 to 5.09%; *P* < 0.001), respectively. However, when the number of cases reached 1 280 000, its effect size decreased to 0.38% (95% CI = 0.10 to 0.67%; *P* = 0.008) and 1.58% (95% CI = 1.06 to 2.11%; *P* < 0.001), respectively. Notably, vaccine rollouts were the most effective measure in later outbreak stages, with the effect size ranging from 1.51% (95% CI = 0.07 to 2.94%; *P* = 0.040) to 3.04% (95% CI = 0.85 to 5.23%; *P* = 0.007).

### Associations between policy measures and case DT vary with government effectiveness

**Figure 3:**
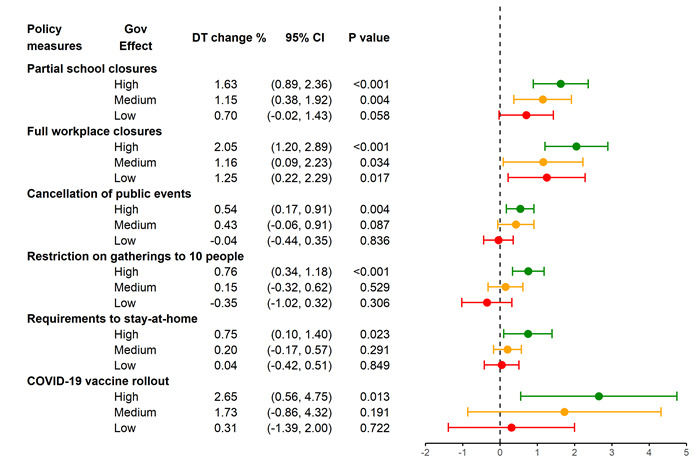
Associations of NPIs and vaccine rollouts with case doubling time (DT), by government effectiveness scores. The first column presents policy variables constructed from the Oxford COVID-19 Government Response Tracker. 137 sample countries were categorised into three equal-sized groups with high, medium and low government effectiveness scores, as shown in the second column. Scores are developed by Worldwide Governance Indicators. The third column is the estimated policy effect, ie, the percentage change in DT for each day of intervention. The length of bar on the right represents the 95% confidence interval; the dot in the middle represents the estimated policy effect.

We further examined whether the effect sizes varied across country groups with high, medium and low government effectiveness scores (**Figure 3**). For countries with high government effectiveness scores, vaccine rollouts were most effective (2.65%; 95% CI = 0.56 to 4.75%; *P* = 0.013), followed by full workplace closures (2.05%; 95% CI = 1.20 to 2.89%; *P* < 0.001). For countries with a low or medium level of government effectiveness, the most effective measure was full workplace closures, which prolonged the DT by 1.25% (95% CI = 0.22 to 2.29%; *P* = 0.017) and 1.16% (95% CI = 0.09 to 2.23%; *P* = 0.034), respectively.

### Sensitivity analysis and model validations

To check robustness of the study results, we re-estimated the model by using alternative specifications (Appendix S8 in the **Online Supplementary Document**). Sensitivity analyses reveal that the estimated policy effects were generally consistent across different model specifications.

**Figure 4:**
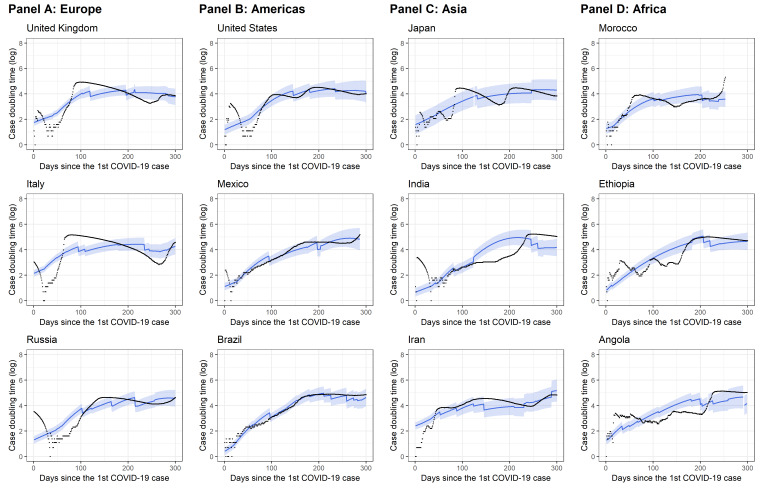
Predicted vs observed trends of case doubling time for the selected countries. Countries selected here represent different geographic locations and had relatively high case numbers. **Panel A.** Europe. **Panel B.** Americas. **Panel C.** Asia. **Panel D.** Africa. The scattered black dots represent observed data. The solid blue line is the predicted case doubling time (log) based on the proposed growth curve model (see Equation (1) in Methods). The shaded area is the 95% confidence interval for the fixed part of the model.

The proposed model was validated using residual diagnostics, a cross-validation method, and through comparison of the predicted with observed DT trajectories for each country. The results are presented in Appendix S9, S10 and S11 of the **Online Supplementary Document**, respectively. **Figure 4** displays the predicted DT for selected countries across four continents that had relatively high case numbers. For all 137 countries, the correlation coefficient of the predicted and observed case DT was 0.81, demonstrating reasonably good prediction accuracy.

## DISCUSSION

Results for the full sample indicate that vaccine rollout is the most effective measure in prolonging case DT, followed by workplace closures and school closures. Closures of workplaces and schools were positively associated with case DT throughout all outbreak stages. For countries with high government effectiveness scores, most containment measures and vaccine rollouts were effective; for countries with lower government effectiveness scores, only workplace closure was consistently associated with prolonged case DT.

The finding regarding the high effectiveness of vaccine rollout accords with clinical evidence that COVID-19 vaccines have high efficacy in reducing the incidence of SARS-CoV-2 infection [29,30]. Prior to the introduction of vaccine rollouts, scholars discovered that lockdown-like NPIs had a larger effect on reducing transmission than public health NPIs, including face covering, did [6,11,13]. By including post-vaccination periods, we determined that the marginal effect of vaccine rollouts appears to outweigh those of NPIs. However, this does not imply that vaccination alone is sufficient to suppress the COVID-19 outbreak. Instead, our interpretation is that vaccine rollouts have the potential to reduce the duration of NPIs for curbing the growth of COVID-19 cases. Simulation studies considering different levels of vaccine uptake and NPIs have suggested that combining vaccination with physical distancing measures will achieve optimal control effects [14,15,17].

Notably, vaccine rollout policies were statistically associated with prolonged DT only in countries with high government effectiveness. A possible explanation is that vaccine coverage was lower in countries with lower levels of government effectiveness. We calculated the number of people who received at least one vaccine dose by using data available on 13 June 2021 for 82 countries, provided by Our World in Data [31]. For countries with high, medium, and low government effectiveness scores, the median coverage rates were 44%, 13%, and 5%, respectively, indicating large differences in coverage. Our recent study indicated that vaccine coverage was statistically associated with reduced case fatality ratios only after coverage reached 8% [32]. We conjectured that to reduce COVID-19 infections in general populations, a minimum coverage rate may be required.

Among the NPIs investigated, workplace closures and school closures were positively associated with case DT throughout all outbreak stages. Their high effectiveness has been demonstrated in cross-country studies that have also used the OxCGRT database and employed different methodologies [8,33-35]. In addition, we observed that the cancellation of public events and the restriction of gatherings to 10 people were effective in earlier outbreak stages; these results were consistent with those from previous studies [34,35]. Scholars have highlighted that earlier implementation of containment measures resulted in greater reductions in SARS-CoV-2 transmission [7,9,10,12]. Our long-term data provided supporting evidence by indicating declining trends in their effectiveness. Population adherence to preventive measures influences the effectiveness of interventions [36]. Pandemic fatigue may partly explain the weaker effects of containment policies in later stages of the outbreak [37].

**Figure 2** does not include the effects of border closures, coordinated information campaigns, or face covering in public places. In fact, these measures were associated with prolonged DT when the number of cases was fewer than 5000 (Table S7.1 in Appendix S7 in the **Online Supplementary Document**). Studies have revealed that during the first infection wave in 2020, border restrictions and risk-communication strategies were highly effective in reducing COVID-19 transmission [9] and that the effects of international travel restrictions were short lived [33]. However, researchers have raised concerns over estimating the effect of mask wearing in public spaces because containment measures have limited public life [8] and because face-covering mandates are an imperfect proxy for mask wearing [38].

Scholars have urged that governments should act to control COVID-19 outbreaks [39]. In this study, higher government effectiveness was associated with prolonged case DT independently of NPIs and vaccination. This suggested that governments played a key role in explaining cross-country variations in case DT. Most importantly, although all containment measures were effective in countries with high government effectiveness scores, only the closure of workplaces was consistently associated with prolonged case DT (and with smaller effect sizes) in countries with lower effectiveness scores. Countries with lower government effectiveness have lower-quality policy formulation and implementation and lack commitment to public policies. As a result, the policy effect estimated per day of intervention was lower in countries where actual implementation was flawed.

The present study has limitations. First, causation between policy measures and attenuation of the spread of COVID-19 is difficult to infer from observational data. Since it is difficult to finely dissect the causes of changes in case DT, our results should be interpreted with caution and not be used as a generalizable guidance for policy making. Randomly implementing different containment or health measures would provide more robust evidence; however, this approach might be infeasible in practice. Second, we used available open data for a comprehensive set of variables, including 11 policy measures, government effectiveness scores, and country characteristics. We also included random intercepts to capture unobserved country-level factors. However, because of the large number of countries included, some variables that were available only for a small number of countries were inevitably excluded. Third, we did not have access to data regarding compliance with containment measures, which is essential for controlling the spread of COVID-19. Poor compliance with containment measures may have led to underestimation of their effect, in which case our results would be conservative. Fourth, data harmonisation is lacking across countries in data input, diagnostics, health care response and outcomes, and reporting standards. This limitation may bias our estimation results. Finally, the case doubling time is a conservative measure and may not be applicable to more complex models that reveal the underlying mechanism of virus transmission, such as the network model.

## CONCLUSIONS

Results for 137 countries indicated that vaccine rollouts, full workplace closures, and partial school closures were relatively effective in attenuating the spread of COVID-19. The effectiveness of vaccine rollouts outweighed that of NPIs, especially in later outbreak stages. However, vaccination was not associated with prolonged case DT in countries with lower levels of government effectiveness, probably due to the low vaccine coverage. Among the NPIs examined, the closure of workplaces was highly effective across all outbreak stages and levels of government effectiveness. Moreover, government effectiveness was vital in the implementation of NPIs; low government effectiveness may undermine the beneficial effects of NPIs. Our findings suggest that mass vaccination is critical for reducing SARS-CoV-2 transmission rates, especially in countries where NPIs are less effective.

## Additional material


Online Supplementary Document
