# Supplementary material for: COVID-19 case doubling time associated with non-pharmaceutical interventions and vaccination: A global experience
Source: J Glob Health. 2021 Sep 4;11:05021. doi: 10.7189/jogh.11.05021 (PMC8442574; doi:10.7189/jogh.11.05021)
Supplement: Online Supplementary Document [file jogh-11-05021-s001.pdf]

# COVID-19 case doubling time associated with non-pharmaceutical interventions and vaccination: a global experience

## SUPPLEMENTARY DOCUMENT


**Appendix S1 Flow chart of the study sample**

**Appendix S2 Sample countries, observed country days and government effectiveness scores**

**Appendix S3 Policy measures based on the Oxford COVID-19 Government Response Tracker (OxCGRT) indicators**

**Appendix S4 Frequency and average duration of policy measures**

**Appendix S5 Summary of model variables**

**Appendix S6 Number of countries implemented the 11 policy measures by calendar date**

**Appendix S7 Results from regressions for COVID-19 case doubling time**

**Appendix S8 Sensitivity analysis: results from alternative specifications**

**Appendix S9 Residual diagnostics**

**Appendix S10 Cross validation of the study results**

**Appendix S11 Predicted versus observed trends of case doubling time for 137 countries**

## Appendix S1 Flow chart of the study sample

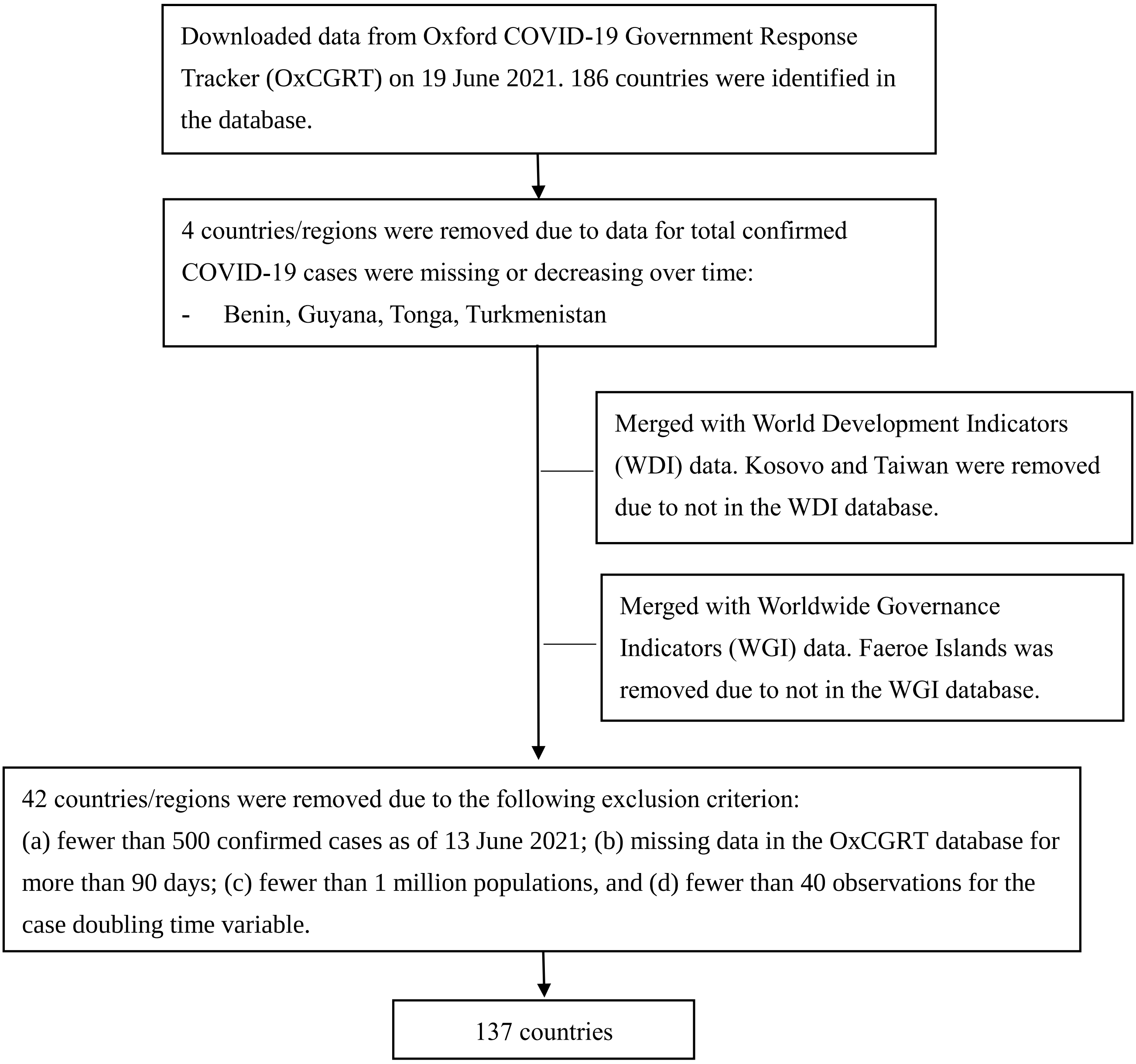


Figure S1. Flow chart of 137 countries analysed in this study

## Appendix S2 Sample countries, observed country days and government effectiveness scores

The study sample is composed of 137 countries over the period of 1st January 2020 – 13rd June, 2021. Observed country days refers to days since the first reported COVID-19 case until the last day after which the case doubling time (the outcome variable) could not be calculated. See Methods for calculation of case doubling time. The government effectiveness scores range from -2.5 to 2.5, with a higher value indicating higher government effectiveness. We applied the latest 2019 scores. For subgroup analysis presented in Figure 3 in the main text, we ranked countries according to their effectiveness scores and divided them into three equal-sized groups: low, medium and high, denoted by L, M, and H, respectively in Table S2.

Table S2. Sample countries, observed country days and government effectiveness scores

| Country | ISO code | Continent | Observed days | Government effectiveness | |
|---|---|---|---|---|---|
| | | | | Score | Level |
| Afghanistan | AFG | Asia | 266 | -1.46 | L |
| Angola | AGO | Africa | 297 | -1.12 | L |
| Albania | ALB | Europe | 312 | -0.06 | M |
| United Arab Emirates | ARE | Asia | 359 | 1.38 | H |
| Argentina | ARG | South America | 356 | -0.09 | M |
| Australia | AUS | Oceania | 183 | 1.57 | H |
| Austria | AUT | Europe | 293 | 1.49 | H |
| Azerbaijan | AZE | Asia | 286 | -0.14 | M |
| Burundi | BDI | Africa | 352 | -1.33 | L |
| Belgium | BEL | Europe | 287 | 1.03 | H |
| Burkina Faso | BFA | Africa | 297 | -0.76 | L |
| Bangladesh | BGD | Asia | 242 | -0.74 | L |
| Bulgaria | BGR | Europe | 310 | 0.34 | H |
| Bahrain | BHR | Asia | 368 | 0.30 | M |
| Bosnia and Herzegovina | BIH | Europe | 286 | -0.63 | L |
| Belarus | BLR | Europe | 313 | -0.18 | M |
| Bolivia | BOL | South America | 315 | -0.70 | L |
| Brazil | BRA | South America | 331 | -0.19 | M |
| Botswana | BWA | Africa | 339 | 0.43 | H |
| Central African Republic | CAF | Africa | 106 | -1.75 | L |
| Canada | CAN | North America | 356 | 1.73 | H |
| Switzerland | CHE | Europe | 286 | 1.95 | H |
| Chile | CHL | South America | 348 | 1.06 | H |
| Cote d'Ivoire | CIV | Africa | 297 | -0.48 | M |

| | | | | | |
|---|---|---|---|---|---|
| Cameroon | CMR | Africa | 370 | -0.81 | L |
| Democratic Republic of Congo | COD | Africa | 295 | -1.63 | L |
| Congo | COG | Africa | 274 | -1.39 | L |
| Colombia | COL | South America | 316 | 0.07 | M |
| Costa Rica | CRI | North America | 304 | 0.42 | H |
| Czech Republic | CZE | Europe | 316 | 0.89 | H |
| Germany | DEU | Europe | 346 | 1.59 | H |
| Denmark | DNK | Europe | 301 | 1.94 | H |
| Dominican Republic | DOM | North America | 288 | -0.36 | M |
| Algeria | DZA | Africa | 264 | -0.52 | M |
| Ecuador | ECU | South America | 314 | -0.40 | M |
| Egypt | EGY | Africa | 321 | -0.42 | M |
| Spain | ESP | Europe | 331 | 1.00 | H |
| Estonia | EST | Europe | 367 | 1.17 | H |
| Ethiopia | ETH | Africa | 316 | -0.63 | L |
| Finland | FIN | Europe | 374 | 1.93 | H |
| France | FRA | Europe | 362 | 1.38 | H |
| Gabon | GAB | Africa | 335 | -0.90 | L |
| United Kingdom | GBR | Europe | 331 | 1.44 | H |
| Georgia | GEO | Asia | 288 | 0.83 | H |
| Ghana | GHA | Africa | 218 | -0.21 | M |
| Guinea | GIN | Africa | 228 | -0.78 | L |
| Gambia | GMB | Africa | 168 | -0.63 | L |
| Greece | GRC | Europe | 377 | 0.41 | H |
| Guatemala | GTM | North America | 290 | -0.68 | L |
| Honduras | HND | North America | 301 | -0.61 | L |
| Croatia | HRV | Europe | 294 | 0.41 | H |
| Haiti | HTI | North America | 169 | -2.02 | L |
| Hungary | HUN | Europe | 355 | 0.50 | H |
| Indonesia | IDN | Asia | 326 | 0.18 | M |
| India | IND | Asia | 443 | 0.17 | M |
| Ireland | IRL | Europe | 314 | 1.28 | H |
| Iran | IRN | Asia | 362 | -0.55 | M |
| Iraq | IRQ | Asia | 336 | -1.34 | L |
| Israel | ISR | Asia | 314 | 1.33 | H |
| Italy | ITA | Europe | 336 | 0.46 | H |
| Jamaica | JAM | North America | 358 | 0.50 | H |
| Jordan | JOR | Asia | 347 | 0.10 | M |

| | | | | | |
|---|---|---|---|---|---|
| Japan | JPN | Asia | 374 | 1.59 | H |
| Kazakhstan | KAZ | Asia | 320 | 0.12 | M |
| Kenya | KEN | Africa | 267 | -0.38 | M |
| Kyrgyz Republic | KGZ | Asia | 221 | -0.68 | L |
| Cambodia | KHM | Asia | 469 | -0.58 | L |
| South Korea | KOR | Asia | 366 | 1.38 | H |
| Kuwait | KWT | Asia | 342 | 0.02 | M |
| Laos | LAO | Asia | 406 | -0.78 | L |
| Lebanon | LBN | Asia | 335 | -0.83 | L |
| Liberia | LBR | Africa | 161 | -1.38 | L |
| Libya | LBY | Africa | 272 | -1.92 | L |
| Sri Lanka | LKA | Asia | 462 | -0.11 | M |
| Lesotho | LSO | Africa | 242 | -0.83 | L |
| Lithuania | LTU | Europe | 305 | 1.04 | H |
| Latvia | LVA | Europe | 338 | 1.11 | H |
| Morocco | MAR | Africa | 253 | -0.12 | M |
| Moldova | MDA | Europe | 282 | -0.38 | M |
| Madagascar | MDG | Africa | 354 | -1.14 | L |
| Mexico | MEX | North America | 287 | -0.16 | M |
| Mali | MLI | Africa | 281 | -1.06 | L |
| Myanmar | MMR | Asia | 236 | -1.15 | L |
| Mongolia | MNG | Asia | 418 | -0.19 | M |
| Mozambique | MOZ | Africa | 312 | -0.82 | L |
| Mauritania | MRT | Africa | 270 | -0.50 | L |
| Mauritius | MUS | Africa | 372 | 0.87 | H |
| Malawi | MWI | Africa | 290 | -0.75 | L |
| Malaysia | MYS | Asia | 420 | 1.00 | H |
| Namibia | NAM | Africa | 311 | 0.10 | M |
| Niger | NER | Africa | 277 | -0.80 | L |
| Nigeria | NGA | Africa | 302 | -1.09 | L |
| Nicaragua | NIC | North America | 137 | -0.77 | L |
| Netherlands | NLD | Europe | 314 | 1.80 | H |
| Norway | NOR | Europe | 344 | 1.86 | H |
| Nepal | NPL | Asia | 407 | -1.05 | L |
| New Zealand | NZL | Oceania | 46 | 1.67 | H |
| Oman | OMN | Asia | 259 | 0.26 | M |
| Pakistan | PAK | Asia | 305 | -0.68 | L |
| Panama | PAN | North America | 279 | 0.07 | M |

| | | | | | |
|---|---|---|---|---|---|
| Peru | PER | South America | 294 | -0.07 | M |
| Philippines | PHL | Asia | 416 | 0.05 | M |
| Papua New Guinea | PNG | Oceania | 386 | -0.81 | L |
| Poland | POL | Europe | 320 | 0.60 | H |
| Portugal | PRT | Europe | 308 | 1.15 | H |
| Paraguay | PRY | South America | 380 | -0.53 | M |
| Qatar | QAT | Asia | 151 | 0.71 | H |
| Romania | ROU | Europe | 289 | -0.28 | M |
| Russia | RUS | Europe | 316 | 0.15 | M |
| Rwanda | RWA | Africa | 307 | 0.19 | M |
| Saudi Arabia | SAU | Asia | 133 | 0.31 | M |
| Senegal | SEN | Africa | 303 | -0.06 | M |
| Singapore | SGP | Asia | 122 | 2.22 | H |
| Sierra Leone | SLE | Africa | 182 | -1.13 | L |
| El Salvador | SLV | North America | 249 | -0.47 | M |
| Serbia | SRB | Europe | 310 | 0.02 | M |
| Slovak Republic | SVK | Europe | 306 | 0.67 | H |
| Slovenia | SVN | Europe | 306 | 1.08 | H |
| Sweden | SWE | Europe | 355 | 1.83 | H |
| Eswatini | SWZ | Africa | 293 | -0.68 | L |
| Chad | TCD | Africa | 296 | -1.57 | L |
| Togo | TGO | Africa | 358 | -0.92 | L |
| Thailand | THA | Asia | 467 | 0.36 | H |
| Tajikistan | TJK | Asia | 76 | -1.05 | L |
| Timor-Leste | TLS | Asia | 413 | -0.88 | L |
| Trinidad and Tobago | TTO | North America | 425 | 0.10 | M |
| Tunisia | TUN | Africa | 322 | -0.10 | M |
| Turkey | TUR | Asia | 349 | 0.05 | M |
| Uganda | UGA | Africa | 276 | -0.59 | L |
| Ukraine | UKR | Europe | 311 | -0.30 | M |
| Uruguay | URY | South America | 404 | 0.70 | H |
| United States | USA | North America | 328 | 1.49 | H |
| Uzbekistan | UZB | Asia | 190 | -0.51 | M |
| Vietnam | VNM | Asia | 488 | 0.04 | M |
| South Africa | ZAF | Africa | 286 | 0.37 | H |
| Zambia | ZMB | Africa | 322 | -0.68 | L |
| Zimbabwe | ZWE | Africa | 295 | -1.21 | L |

## Appendix S3 Policy measures based on the Oxford COVID-19 Government Response Tracker (OxCGRT) indicators

Table S3 provides the definition of policy measures used in this study and the corresponding OxCGRT indicators. We focused on interventions that were actually enforced; thus for most OxCGRT indicators, degree of 0 and 1 were treated as the reference group, i.e. no measures. In addition, we combined the highest two degrees for stay-at-home requirements, testing policies, and face covering. For vaccine rollout, we combined degrees of 1−5 into a single category because the number of country-days for individual degrees was relatively small.

Table S3. Policy measures based on Oxford COVID-19 Government Response Tracker (OxCGRT) indicators

| OxCGRT indicators | | Policy measures in this study |
|---|---|---|
| Degree | **School closures** | |
| 0 | No measures | No measures |
| 1 | Recommend closing | No measures |
| 2 | Require closing (some levels or categories) | Partial closures |
| 3 | Require closing (all levels) | Full closures |
| Degree | **Workplace closures, or work-from-home** | |
| 0 | No measures | No measures |
| 1 | Recommend closing | No measures |
| 2 | Require closing (some sectors or categories) | Partial closures |
| 3 | Require closing (all-but-essential workplaces) | Full closures |
| Degree | **Cancellation of public events** | |
| 0 | No measures | No measures |
| 1 | Recommend cancelling | No measures |
| 2 | Require cancelling | Requirement of cancellation |
| Degree | **Restrictions on gathering size** | |
| 0 | No restriction | No limits or up to 1000 people |
| 1 | Restrictions on gatherings above 1000 people | No limits or up to 1000 people |
| 2 | Restrictions on gatherings between 101-1000 people | No limits or up to 1000 people |
| 3 | Restrictions on gatherings between 11-100 people | Up to 100 people |
| 4 | Restrictions on gatherings of 10 people or less | Up to 10 people |

| Degree | **Requirements to stay-at-home** | |
|---|---|---|
| 0 | No measures | No measures |
| 1 | Recommend not leaving house | No measures |
| 2 | Require not leaving house with exceptions for daily exercise, grocery shopping and essential trips | Required, with exceptions |
| 3 | Require not leaving house with minimal exceptions | Required, with exceptions |
| Degree | **Restrictions on international travel** | |
| 0 | No measures | No international travel bans |
| 1 | Screening | No international travel bans |
| 2 | Quarantine arrivals from high-risk regions | No international travel bans |
| 3 | Ban on arrivals from some regions | Bans on arrivals from some regions |
| 4 | Ban on all regions or total border closure | Bans on arrivals from all regions |
| Degree | **Public information campaigns** | |
| 0 | No campaigns | No or limited campaigns |
| 1 | Public officials urging caution about COVID-19 | No or limited campaigns |
| 2 | Coordinated public information campaign | Coordinated public campaigns |
| Degree | **Testing policy** | |
| 0 | No testing policy | No or limited testing |
| 1 | Only those who meet specific criteria and have symptoms | No or limited testing |
| 2 | Testing of anyone with COVID-19 symptoms | Widespread testing |
| 3 | Open public testing | Widespread testing |
| Degree | **Contact tracing** | |
| 0 | No contact tracing | No or limited contact tracing |
| 1 | Contact tracing not done for all cases | No or limited contact tracing |
| 2 | Contact tracing done for all identified cases | Comprehensive contact tracing |
| Degree | **Face covering** | |
| 0 | No policy | No measures |
| 1 | Recommended | No measures |
| 2 | Required in some specified shared/public spaces | Required in some places |
| 3 | Required in all shared/public spaces | Required in all places |
| 4 | Required outside the home at all times | Required in all places |
| Degree | **Vaccination policy** | |
| 0 | No availability | No vaccination |

| | | |
|---|---|---|
| 1 | Availability for one of the following: key workers/clinically vulnerable groups/elderly groups | Vaccine rollout |
| 2 | Availability for two of the following: key workers/clinically vulnerable groups/elderly groups | Vaccine rollout |
| 3 | Availability for all of the following: key workers/clinically vulnerable groups/elderly groups | Vaccine rollout |
| 4 | Availability for all three plus partial additional availability | Vaccine rollout |
| 5 | Universal availability | Vaccine rollout |

Note: Data for OxCGRT indicators were retrieved from https://COVIDtracker.bsg.ox.ac.uk/,
on 19 June, 2021. Data source: Hale Thomas, Sam Webster, Anna Petherick, Toby Phillips, and Beatriz Kira (2021). Oxford COVID-19 Government Response Tracker, Blavatnik School of Government.

## Appendix S4 Frequency and average duration of policy measures

Figures in Table S4 were calculated based on 137 sample countries over the period of 1st January 2020 – 13rd June, 2021. The most frequently adopted policy was "workplace closures for some sectors or categories" (355 times), followed by "requirements to stay-at-home with exceptions for daily exercise, grocery shopping and essential trips" (289 times), and school closures at all levels (272 times). With respect to average duration, coordinated public information campaigns had the longest days (358 days), followed by comprehensive contact tracing (218 days) and cancellation of public events (168 days).

Table S4. Frequency and average duration of COVID-19 policy measures

| | OxCGRT policy indicator | Total country days | Frequency of adoption | Average number of days |
|---|---|---|---|---|
| Degree | **School closures** | | | |
| 1 | Recommend closing | 14477 | 201 | 72.02 |
| 2 | Require closing (some levels or categories) | 15468 | 266 | 58.15 |
| 3 | Require closing (all levels) | 26856 | 272 | 98.74 |
| Degree | **Workplace closures, or work-from-home** | | | |
| 1 | Recommend closing | 12841 | 187 | 68.67 |
| 2 | Require closing (some sectors or categories) | 31102 | 355 | 87.61 |
| 3 | Require closing (all-but-essential workplaces) | 9832 | 216 | 45.52 |
| Degree | **Cancellation of public events** | | | |
| 1 | Recommend cancelling | 12400 | 202 | 61.39 |
| 2 | Require cancelling | 44159 | 263 | 167.90 |
| Degree | **Restrictions on gathering size** | | | |
| 1 | Restrictions on gatherings above 1000 people | 1687 | 43 | 39.23 |
| 2 | Restrictions on gatherings between 101-1000 people | 6062 | 129 | 46.99 |
| 3 | Restrictions on gatherings between 11-100 people | 19109 | 264 | 72.38 |
| 4 | Restrictions on gatherings of 10 people or less | 28992 | 248 | 116.90 |
| Degree | **Requirements to stay-at-home** | | | |
| 1 | Recommend not leaving house | 18622 | 256 | 72.74 |
| 2 | Require not leaving house with exceptions for daily exercise, grocery shopping and essential trips | 24356 | 289 | 84.28 |
| 3 | Require not leaving house with minimal exceptions | 3546 | 84 | 42.21 |
| Degree | **Restrictions on international travel** | | | |
| 1 | Screening | 10674 | 175 | 60.99 |

| | | | | |
|---|---|---|---|---|
| 2 | Quarantine arrivals from high-risk regions | 13578 | 226 | 60.08 |
| 3 | Ban on arrivals from some regions | 21761 | 257 | 84.67 |
| 4 | Ban on all regions or total border closure | 18685 | 169 | 110.56 |
| Degree | **Public information campaigns** | | | |
| 1 | Public officials urging caution about COVID-19 | 4448 | 89 | 49.98 |
| 2 | Coordinated public information campaign | 61253 | 171 | 358.20 |
| Degree | **Testing policy** | | | |
| 1 | Only those who meet specific criteria and have symptoms | 21365 | 163 | 131.07 |
| 2 | Testing of anyone with COVID-19 symptoms | 26265 | 163 | 161.14 |
| 3 | Open public testing | 16210 | 118 | 137.37 |
| Degree | **Contact tracing** | | | |
| 1 | Contact tracing not done for all cases | 23557 | 164 | 143.64 |
| 2 | Contact tracing done for all identified cases | 34397 | 158 | 217.70 |
| Degree | **Face covering** | | | |
| 1 | Recommended | 4071 | 65 | 62.63 |
| 2 | Required in some specified shared/public spaces | 9653 | 115 | 83.94 |
| 3 | Required in all shared/public spaces | 27124 | 199 | 136.30 |
| 4 | Required outside the home at all times | 13777 | 112 | 123.01 |
| Degree | **Vaccination policy** | | | |
| 1 | Availability for one of the following: key workers/clinically vulnerable groups/elderly groups | 2639 | 86 | 30.69 |
| 2 | Availability for two of the following: key workers/clinically vulnerable groups/elderly groups | 4693 | 102 | 46.01 |
| 3 | Availability for all of the following: key workers/clinically vulnerable groups/elderly groups | 5250 | 114 | 46.05 |
| 4 | Availability for all three plus partial additional availability | 2533 | 85 | 29.80 |
| 5 | Universal availability | 1280 | 30 | 42.67 |

## Appendix S5 Summary of model variables

Table S5 summarises model variables based on the full sample, which had 4,2012 country days. The table excludes all the squared terms in the Equation (1) in the main text. The mean log of case doubling time was 3.8. The mean number of weeks from the first reported death to the earliest intervention was −7.26, indicating that countries generally adopted at least one policy measure before the first death was reported.

Table S5. Descriptive statistics of model variables

| **Variables** | **Mean** | **SD** | **Min.** | **Max.** |
|---|---|---|---|---|
| Case doubling time (days, log) | 3.80 | 1.14 | 0 | 6.05 |
| Number of days since the first confirmed case | 163.50 | 101.68 | 1 | 488 |
| Number of days since the most recent implementation at date *t* (0 if no policy at date *t*) | | | | |
| Partial school closures | 10.61 | 26.49 | 0 | 207 |
| Full school closures | 45.00 | 69.31 | 0 | 349 |
| Partial workplace closures | 38.55 | 60.95 | 0 | 357 |
| Full workplace closures | 5.19 | 18.50 | 0 | 266 |
| Cancellation of public events | 77.83 | 85.93 | 0 | 418 |
| Restriction on gatherings to 100 people | 21.94 | 49.53 | 0 | 358 |
| Restriction on gatherings to 10 people | 35.42 | 62.81 | 0 | 341 |
| Stay-at-home requirements | 26.99 | 50.50 | 0 | 328 |
| Bans on arrivals from some regions | 25.46 | 54.39 | 0 | 325 |
| Bans on arrivals from all regions | 28.56 | 55.22 | 0 | 416 |
| Coordinated public campaigns | 151.80 | 104.21 | 0 | 487 |
| Widespread testing | 66.34 | 83.26 | 0 | 408 |
| Comprehensive contact tracing | 72.61 | 96.60 | 0 | 452 |
| Face covering required in some places | 13.48 | 41.90 | 0 | 328 |
| Face covering required in all places | 51.42 | 71.39 | 0 | 373 |
| COVID-19 vaccine rollout | 0.90 | 5.79 | 0 | 113 |
| Government effectiveness scores (-2.5 – 2.5) | 0.04 | 0.93 | -2.02 | 2.22 |
| Number of weeks from first death to the earliest policy | -7.26 | 11.29 | -61.14 | 1 |
| Population (log) | 16.58 | 1.41 | 13.96 | 21.05 |
| Population aged 65 and above (%) | 9.63 | 6.85 | 1.16 | 28.00 |
| Number of people per squared kilometre of land area (hundreds) | 1.74 | 4.82 | 0.02 | 79.53 |
| Universal Health Coverage service coverage index (0–100) | 65.38 | 15.09 | 28 | 89 |

| | | | | |
|---|---|---|---|---|
| GDP per capita adjusted by purchasing power parity (current international dollars, log) | 9.43 | 1.15 | 6.62 | 11.49 |
| Death caused by communicable diseases and maternal/prenatal/nutrition conditions (%) | 21.62 | 20.28 | 1.3 | 65.3 |
| Europe (=1) | 0.26 | 0.44 | 0 | 1 |
| Africa (=1) | 0.28 | 0.45 | 0 | 1 |
| Asia (=1) | 0.29 | 0.45 | 0 | 1 |
| North America (=1) | 0.09 | 0.29 | 0 | 1 |
| South America (=1) | 0.07 | 0.26 | 0 | 1 |
| Oceania (=1) | 0.01 | 0.12 | 0 | 1 |

Note: N=42,102 country days. Data for government effectiveness scores were retrieved from Worldwide Governance Indicator Database at http://info.worldbank.org/governance/wgi/#home. Data for country characteristics were collected from World Development Indicators (WDI) at https://datatopics.worldbank.org/world-development-indicators/. The latest year WDI were available for most countries was: year 2019 for population size, percentage of old people, and GDP per capita; year 2018 for population density; year 2017 for University Health Coverage index, and year 2016 for cause of death. When a country had missing data in the designated year, we replaced that missing value with the country's recoded value from the previous one or two years.

**Appendix S6 Number of countries implemented the 11 policy measures by calendar date**

Figure S6 illustrates the number of countries out of 137 sample countries that adopted respective policy measures each day from 1 January 2020 to May 31 2021. The X-axis denotes calendar date. For example, Jan20 refers to January 2020, and May21 refers to May 2021. Data for all policy measures were obtained from Oxford COVID-19 Government Response Tracker (OxCGRT) indicators database.

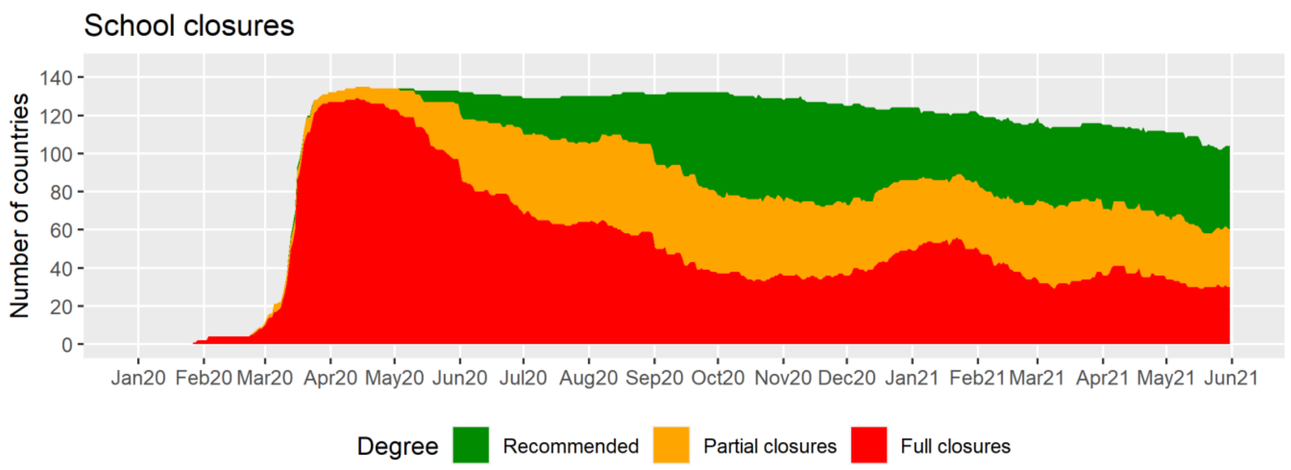


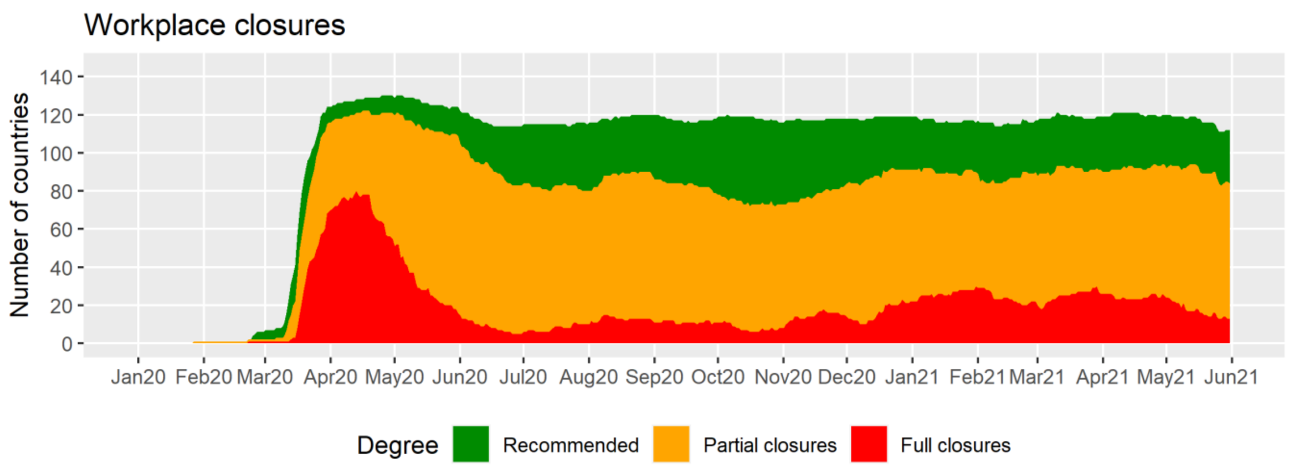

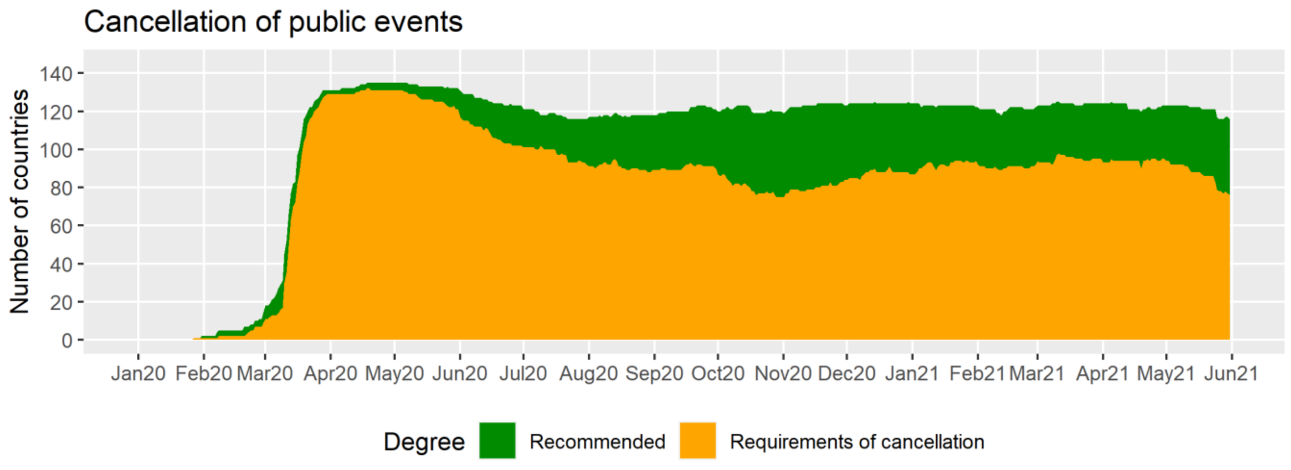
Cancellation of public events
Number of countries
140
120
100
80
60
40
20
0
Jan20 Feb20 Mar20 Apr20 May20 Jun20 Jul20 Aug20 Sep20 Oct20 Nov20 Dec20 Jan21 Feb21 Mar21 Apr21 May21 Jun21
Degree
Recommended
Requirements of cancellation

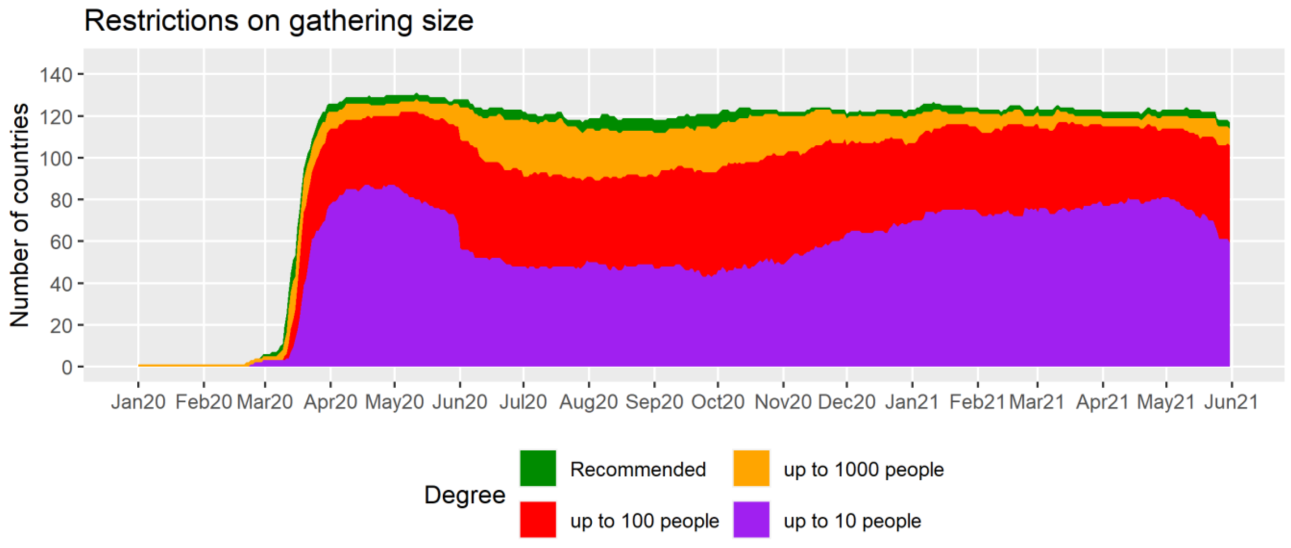
Restrictions on gathering size
Number of countries
140
120
100
80
60
40
20
0
Jan20 Feb20 Mar20 Apr20 May20 Jun20 Jul20 Aug20 Sep20 Oct20 Nov20 Dec20 Jan21 Feb21 Mar21 Apr21 May21 Jun21
Degree
Recommended
up to 1000 people
up to 100 people
up to 10 people

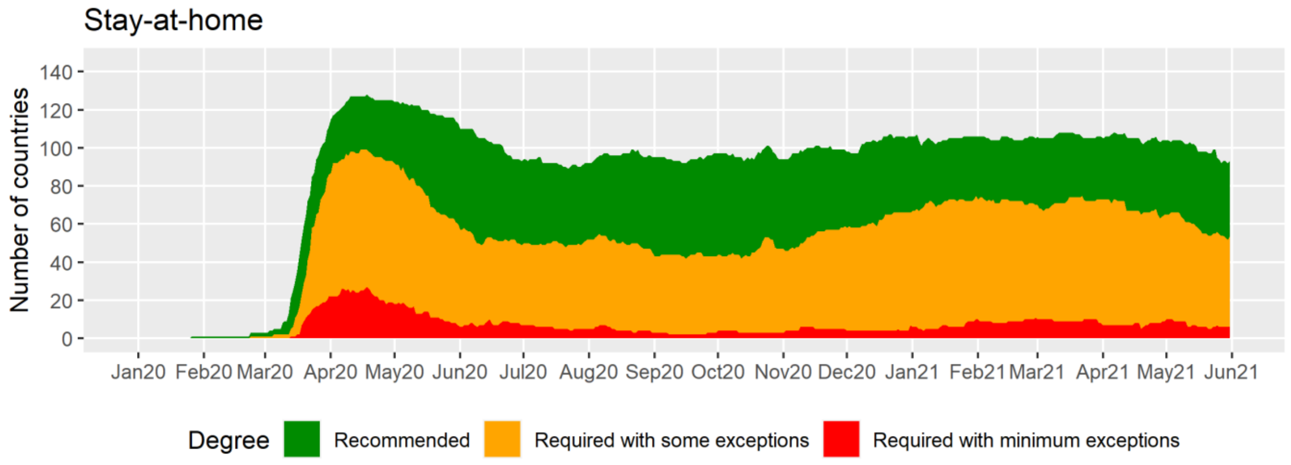
Stay-at-home
Number of countries
140
120
100
80
60
40
20
0
Jan20 Feb20 Mar20 Apr20 May20 Jun20 Jul20 Aug20 Sep20 Oct20 Nov20 Dec20 Jan21 Feb21 Mar21 Apr21 May21 Jun21
Degree
Recommended
Required with some exceptions
Required with minimum exceptions

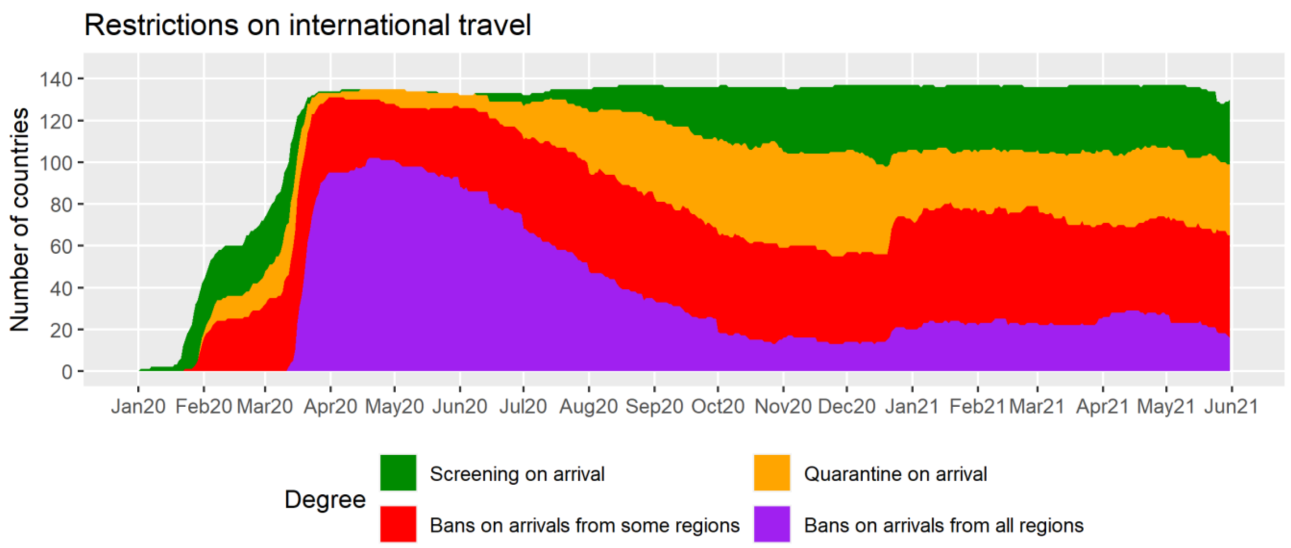
Restrictions on international travel
Number of countries
140
120
100
80
60
40
20
0
Jan20 Feb20 Mar20 Apr20 May20 Jun20 Jul20 Aug20 Sep20 Oct20 Nov20 Dec20 Jan21 Feb21 Mar21 Apr21 May21 Jun21
Degree
Screening on arrival
Quarantine on arrival
Bans on arrivals from some regions
Bans on arrivals from all regions

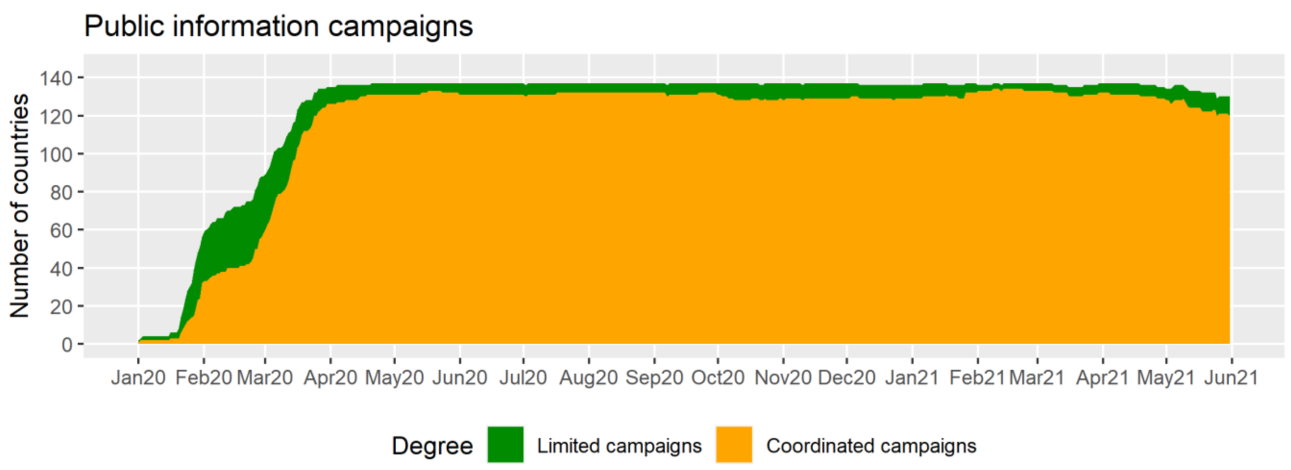
Public information campaigns
Number of countries
140
120
100
80
60
40
20
0
Jan20 Feb20 Mar20 Apr20 May20 Jun20 Jul20 Aug20 Sep20 Oct20 Nov20 Dec20 Jan21 Feb21 Mar21 Apr21 May21 Jun21
Degree
Limited campaigns
Coordinated campaigns

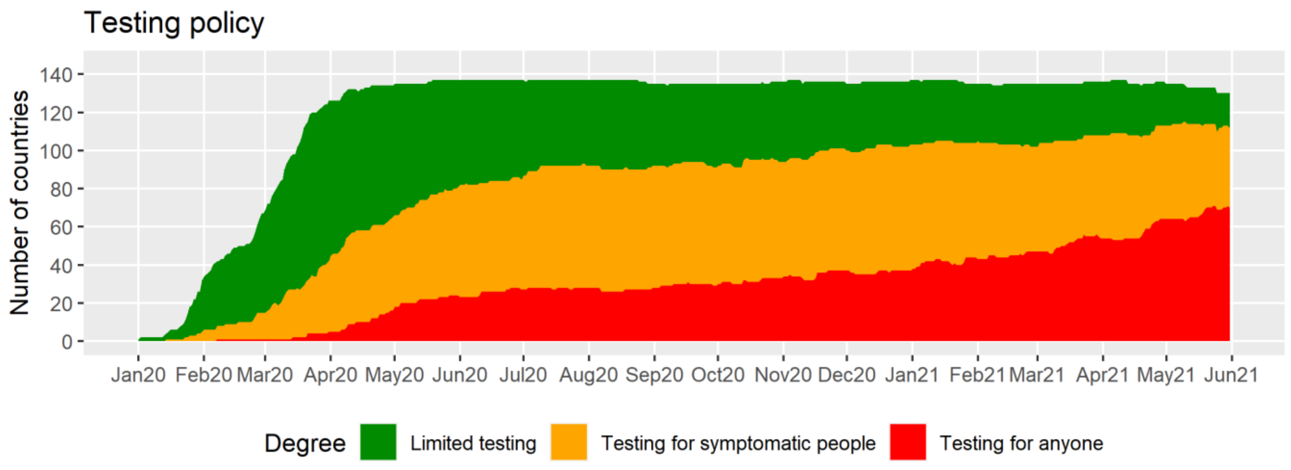
Testing policy
Number of countries
140
120
100
80
60
40
20
0
Jan20 Feb20 Mar20 Apr20 May20 Jun20 Jul20 Aug20 Sep20 Oct20 Nov20 Dec20 Jan21 Feb21 Mar21 Apr21 May21 Jun21
Degree
Limited testing
Testing for symptomatic people
Testing for anyone

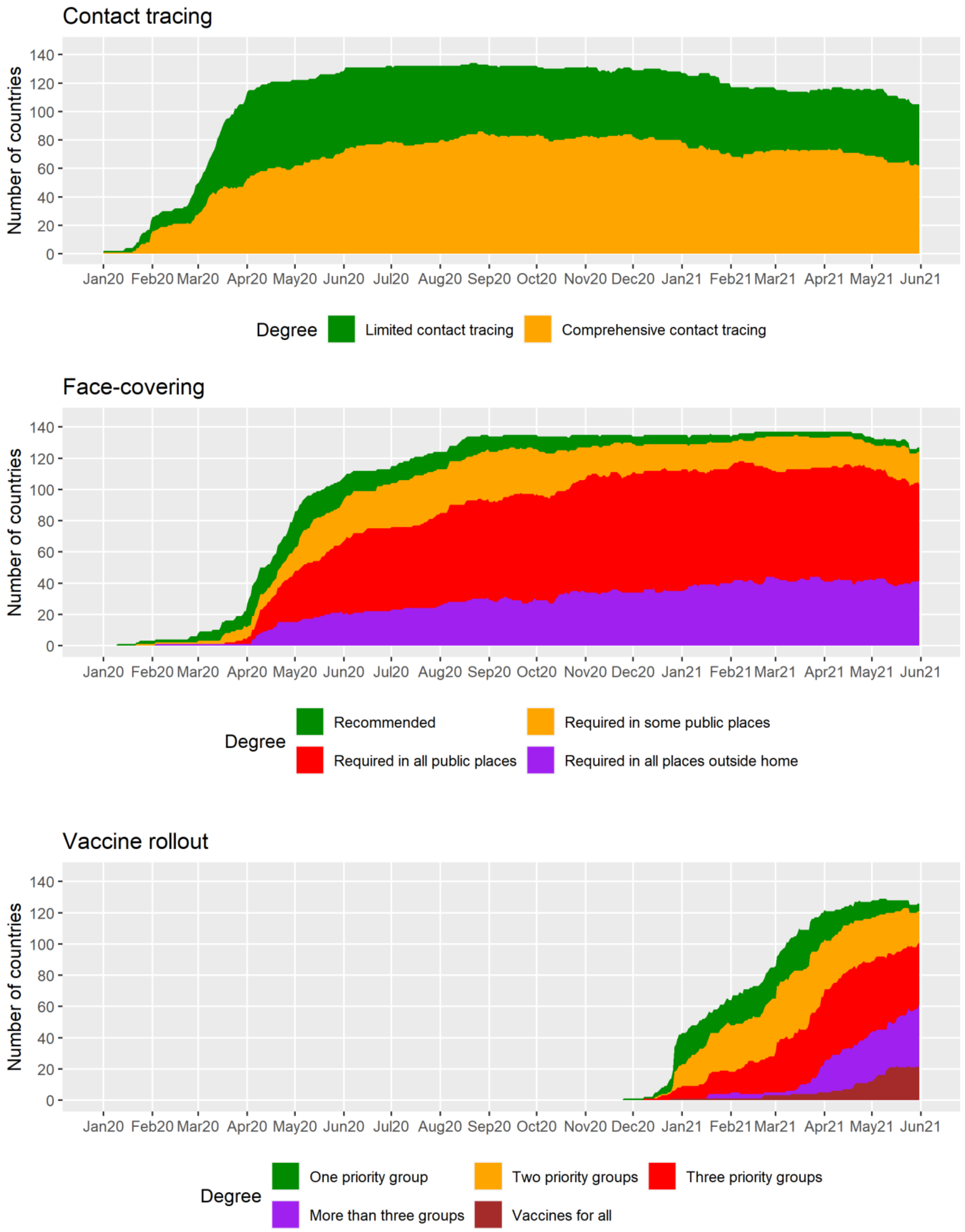


Figure S6. Number of countries implemented the 11 policy measures by calendar date.

## Appendix S7 Results from regressions for COVID-19 case doubling time

Table S7.1 Results from regressions based on the full sample and by outbreak stages

| Y: case doubling time (in log days) | Full sample | Subsample regressions by outbreak stages (cases) | | | | |
|---|---|---|---|---|---|---|
| | | <5,000 | <20,000 | <80,000 | <320,000 | <1,280,000 |
| Predictors | Coef. | Coef. | Coef. | Coef. | Coef. | Coef. |
| No. of days since the most recent implementation | | | | | | |
| Partial school closures | 0.0138*** | 0.0008 | 0.0070* | 0.0120*** | 0.0127*** | 0.0132*** |
| Full school closures | 0.0040** | 0.0077* | 0.0061* | 0.0051* | 0.0037* | 0.0038** |
| Partial workplace closures | 0.0009 | 0.0011 | -0.0008 | -0.0012 | 0.0005 | 0.0003 |
| Full workplace closures | 0.0141*** | 0.0384*** | 0.0312*** | 0.0227*** | 0.0248*** | 0.0158*** |
| Cancellation of public events | 0.0021 | 0.0058** | 0.0039 | 0.0019 | 0.0021 | 0.0017 |
| Restriction on gatherings to 100 people | 0.0028 | -0.0039 | -0.0021 | 0.0014 | 0.0021 | 0.0023 |
| Restriction on gatherings to 10 people | 0.003 | 0.0074 | 0.0077* | 0.0046 | 0.0050** | 0.0041* |
| Stay-at-home requirements | 0.0015 | 0.0056* | 0.0012 | 0.0018 | 0.002 | 0.0018 |
| Bans on arrivals from some regions | -0.0004 | 0.0033 | 0.0022 | 0.0021 | 0.0013 | 0.0002 |
| Bans on arrivals from all regions | 0.0018 | 0.0064* | 0.002 | 0.003 | 0.0031 | 0.0021 |
| Coordinated public campaigns | 0.0039 | 0.0086** | 0.0037 | 0.0036 | 0.0034 | 0.0038 |
| Widespread testing | -0.0023 | -0.0022 | -0.0022 | -0.0029 | -0.002 | -0.0021 |
| Comprehensive contact tracing | -0.0019 | -0.0017 | 0.0000 | -0.0003 | -0.0022 | -0.0024 |
| Face covering required in some places | -0.0003 | -0.004 | 0.0001 | 0.0021 | 0.0005 | 0.0000 |
| Face covering required in all places | -0.0001 | 0.0066* | 0.0011 | 0.0012 | 0.0019 | 0.001 |
| COVID-19 vaccine rollout | 0.0196** | 0.0176 | 0.019 | 0.0304** | 0.0151* | 0.0208** |
| Government effectiveness scores (-25 – 25) | 0.0226* | 0.0257* | 0.0326** | 0.0319** | 0.0281** | 0.0244* |
| No. of weeks from 1st death to earliest policy | -0.014 | -0.0164* | -0.0125 | -0.0129 | -0.015 | -0.0143 |
| Population (log) | -0.1607*** | -0.1031* | -0.1609*** | -0.1668*** | -0.1634*** | -0.1553*** |
| Population aged 65 and above (%) | 0.0344 | 0.0261 | 0.0325 | 0.038 | 0.0384* | 0.034 |
| No. of people per $km^2$ of land area | -0.0083* | -0.0038 | -0.0073 | -0.0095* | -0.0086* | -0.0081* |
| UHC service coverage index (0–100) | 0.0046 | -0.0062 | -0.0026 | -0.0021 | 0.0043 | 0.0044 |
| GDP per capita adjusted by PPP (log) | -0.2255* | -0.1861 | -0.3054** | -0.2943** | -0.2716* | -0.2344* |
| Death caused by communicable diseases (%) | 0.0024 | -0.0042 | -0.0048 | -0.0021 | 0.0026 | 0.0024 |
| Africa (=1) | 0.0468 | 0.2711 | 0.2351 | 0.0913 | 0.0039 | 0.0361 |
| Asia (=1) | 0.2627 | 0.3215 | 0.4012 | 0.3197 | 0.2443 | 0.2534 |
| North America (=1) | -0.3071 | 0.073 | -0.1478 | -0.3013 | -0.4304 | -0.3499 |
| South America (=1) | -0.3705 | -0.0528 | -0.1231 | -0.2351 | -0.4141 | -0.4043 |
| Oceania (=1) | 0.5917* | 0.4409* | 0.4683* | 0.4591* | 0.5520* | 0.6089* |
| Number of observations (country days) | 42102 | 17353 | 24762 | 31658 | 37935 | 40804 |

Note: UHC=universal health coverage; GDP=gross domestic product. Note: All models included the time trend, quadratic terms of time and policy variables, and country-specific random intercepts and random coefficients of time. Country-clustered robust standard errors were used. Legend: * p<0.05; ** p<0.01; *** p<0.001.

Table S7.2 Results from regressions by levels of government effectiveness

| Y: case doubling time (in log days) | **Subsample regressions by government effectiveness** | | |
|---|---|---|---|
| | **High** | **Medium** | **Low** |
| Predictors | Coef. | Coef. | Coef. |
| No. of days since the most recent implementation | | | |
| Partial school closures | 0.0163*** | 0.0115** | 0.007 |
| Full school closures | 0.0095* | 0.0008 | 0.0036 |
| Partial workplace closures | 0.0026 | -0.001 | 0.0024 |
| Full workplace closures | 0.0205*** | 0.0116* | 0.0125* |
| Cancellation of public events | 0.0054** | 0.0043 | -0.0004 |
| Restriction on gatherings to 100 people | 0.0009 | 0.0028 | 0.0024 |
| Restriction on gatherings to 10 people | 0.0076*** | 0.0015 | -0.0035 |
| Stay-at-home requirements | 0.0075* | 0.002 | 0.0004 |
| Bans on arrivals from some regions | 0.0022 | -0.0028 | 0.0009 |
| Bans on arrivals from all regions | 0.0065* | -0.0002 | 0.0009 |
| Coordinated public campaigns | 0.0042 | 0.0016 | -0.0017 |
| Widespread testing | -0.0036 | 0.0016 | -0.005 |
| Comprehensive contact tracing | -0.0008 | 0.0006 | -0.0021 |
| Face covering required in some places | 0.0001 | -0.0021 | 0.0086 |
| Face covering required in all places | 0.0000 | -0.0008 | 0.003 |
| COVID-19 vaccine rollout | 0.0265* | 0.0173 | 0.0031 |
| Government effectiveness scores (-25 – 25) | 0.0041 | -0.0295 | 0.0159 |
| No. of weeks from 1st death to earliest policy | 0.0840** | -0.0028 | -0.0191* |
| Population (log) | -0.2616*** | -0.0941** | -0.091 |
| Population aged 65 and above (%) | 0.0217 | 0.0435* | -0.0748 |
| No. of people per $km^2$ of land area | 0.0029 | -0.0057 | -0.0317 |
| UHC service coverage index (0–100) | 0.0236 | 0.0051 | 0.0032 |
| GDP per capita adjusted by PPP (log) | -0.2078 | 0.0587 | -0.1911 |
| Death caused by communicable diseases (%) | -0.0164 | 0.0024 | 0.0064 |
| Africa (=1) | 0.796 | 0.4624 | -1.723 |
| Asia (=1) | 0.5214* | 0.4272 | -1.0284 |
| North America (=1) | -0.4599 | 0.0007 | -1.5287 |
| South America (=1) | -0.1965 | -0.2 | -1.5947 |
| Oceania (=1) | 0.621 | (omitted) | -1.1063 |
| Number of countries | 46 | 45 | 46 |
| Number of observations | 14661 | 14294 | 13147 |

Note: UHC=universal health coverage; GDP=gross domestic product. PPP=purchasing power parity. Note: All models included the time trend, quadratic terms of time and policy variables, and country-specific random intercepts and random coefficients of time. Country-clustered robust standard errors were used. Legend: * p<0.05; ** p<0.01; *** p<0.001

## Appendix S8 Sensitivity analysis: results from alternative specifications

Sensitivity analysis was conducted to check the robustness of the study results. We examined the collinearity by calculating the correlation coefficient (cc) of each pair of policy variables, and excluded those that had a cc greater than 0.4. As Table S8.1 shows (figures in bold), cancellation of public events (ID=5) was highly correlated with full school closures (0.42), partial workplace closures (0.41) and restriction on gatherings to 10 people (0.44). Coordinated public campaigns (ID=11) was highly correlated with widespread testing (0.46), comprehensive contact tracing (0.49), and face covering required in all places (0.46). Therefore, we excluded these two policy measures one by one from the main model. The results are presented under the heading of Model S1 and Model S2 in Table S8.2. We found that results from alternative specifications are generally consistent with those from the main model, except that in Model S1 "Restriction on gatherings to 10 people" became positively associated with prolonged case doubling time.

Table S8.1 Pairwise correlation coefficients between policy variables

| Policy measures | ID | 1 | 2 | 3 | 4 | 5 | 6 | 7 | 8 | 9 | 10 | 11 | 12 | 13 | 14 | 15 | 16 |
|---|---|---|---|---|---|---|---|---|---|---|---|---|---|---|---|---|---|
| Partial school closures | **1** | 1.00 | | | | | | | | | | | | | | | |
| Full school closures | **2** | -0.26 | 1.00 | | | | | | | | | | | | | | |
| Partial workplace closures | **3** | 0.14 | 0.16 | 1.00 | | | | | | | | | | | | | |
| Full workplace closures | **4** | -0.08 | 0.23 | -0.18 | 1.00 | | | | | | | | | | | | |
| Cancellation of public events | **5** | 0.09 | **0.42** | **0.41** | 0.12 | 1.00 | | | | | | | | | | | |
| Restriction on gatherings to 100 people | **6** | 0.09 | 0.00 | -0.07 | -0.05 | 0.15 | 1.00 | | | | | | | | | | |
| Restriction on gatherings to 10 people | **7** | 0.00 | 0.25 | 0.30 | 0.08 | **0.44** | -0.25 | 1.00 | | | | | | | | | |
| Stay-at-home requirements | **8** | -0.01 | 0.28 | 0.13 | 0.25 | 0.36 | 0.20 | 0.21 | 1.00 | | | | | | | | |
| Bans on arrivals from some regions | **9** | 0.01 | -0.08 | 0.11 | 0.00 | 0.13 | -0.07 | 0.18 | -0.01 | 1.00 | | | | | | | |
| Bans on arrivals from all regions | **10** | -0.03 | 0.20 | 0.09 | 0.04 | 0.20 | 0.04 | 0.12 | 0.07 | -0.24 | 1.00 | | | | | | |
| Coordinated public campaigns | **11** | 0.15 | 0.10 | 0.31 | -0.02 | 0.35 | 0.15 | 0.20 | 0.21 | 0.23 | 0.08 | 1.00 | | | | | |
| Widespread testing | **12** | 0.06 | 0.11 | 0.18 | -0.01 | 0.29 | 0.01 | 0.19 | 0.09 | 0.25 | 0.01 | **0.46** | 1.00 | | | | |
| Comprehensive contact tracing | **13** | 0.11 | -0.01 | 0.23 | 0.02 | 0.18 | -0.03 | 0.09 | 0.11 | 0.07 | 0.07 | **0.49** | 0.29 | 1.00 | | | |
| Face covering required in some places | **14** | 0.06 | -0.07 | 0.13 | 0.07 | 0.07 | -0.07 | -0.03 | -0.08 | 0.19 | 0.02 | 0.17 | 0.17 | 0.15 | 1.00 | | |
| Face covering required in all places | **15** | 0.08 | 0.20 | 0.20 | -0.03 | 0.35 | 0.27 | 0.24 | 0.32 | 0.01 | 0.02 | **0.46** | 0.23 | 0.25 | -0.23 | 1.00 | |
| COVID-19 vaccine rollout | **16** | 0.01 | -0.01 | 0.07 | 0.04 | 0.10 | 0.01 | 0.10 | 0.04 | 0.04 | 0.04 | 0.31 | 0.15 | 0.17 | 0.00 | 0.17 | 1.00 |

Table S8.2 Results from alternative model specifications

| Y: case doubling time (in log days) | **Main model** | **Model S1** | **Model S2** |
|---|---|---|---|
| Predictors | Coef. | Coef. | Coef. |
| No. of days since the most recent implementation | | | |
| Partial school closures | 0.0138*** | 0.0141*** | 0.0139*** |
| Full school closures | 0.0040** | 0.0043** | 0.0039** |
| Partial workplace closures | 0.0009 | 0.0015 | 0.001 |
| Full workplace closures | 0.0141*** | 0.0143*** | 0.0143*** |
| Cancellation of public events | 0.0021 | | 0.0021 |
| Restriction on gatherings to 100 people | 0.0028 | 0.0034 | 0.0029 |
| Restriction on gatherings to 10 people | 0.003 | 0.0037** | 0.0029 |
| Stay-at-home requirements | 0.0015 | 0.002 | 0.0014 |
| Bans on arrivals from some regions | -0.0004 | -0.0004 | -0.0002 |
| Bans on arrivals from all regions | 0.0018 | 0.0022 | 0.0019 |
| Coordinated public campaigns | 0.0039 | 0.0042 | |
| Widespread testing | -0.0023 | -0.0021 | -0.002 |
| Comprehensive contact tracing | -0.0019 | -0.002 | -0.0018 |
| Face covering required in some places | -0.0003 | -0.0001 | -0.0005 |
| Face covering required in all places | -0.0001 | 0.0002 | 0.0001 |
| COVID-19 vaccine rollout | 0.0196** | 0.0209** | 0.0196** |
| Government effectiveness scores (-25 – 25) | 0.0226* | 0.0232* | 0.0244** |
| Number of weeks from first death to the earliest policy | -0.014 | -0.0137 | -0.0143* |
| Population (log) | -0.1607*** | -0.1604*** | -0.1699*** |
| Population aged 65 and above (%) | 0.0344 | 0.0339 | 0.0343 |
| Number of people per $km^2$of land area | -0.0083* | -0.0082* | -0.0079* |
| Universal Health Coverage service coverage index (0–100) | 0.0046 | 0.0044 | 0.0043 |
| GDP per capita adjusted by purchasing power parity (log) | -0.2255* | -0.2323* | -0.2362* |
| Death caused by communicable diseases (%) | 0.0024 | 0.002 | 0.0029 |
| Africa (=1) | 0.0468 | 0.0439 | 0.0293 |
| Asia (=1) | 0.2627 | 0.2585 | 0.2781 |
| North America (=1) | -0.3071 | -0.3142 | -0.2977 |
| South America (=1) | -0.3705 | -0.3682 | -0.3554 |
| Oceania (=1) | 0.5917* | 0.5911* | 0.5890* |
| Number of countries | 137 | 137 | 137 |
| Number of observations (country days) | 42102 | 42103 | 42103 |

Note: All models included the time trend, quadratic terms of time and policy variables, and country-specific random intercepts and random coefficients of time. Country-clustered robust standard errors were used. Legend: * p<0.05; ** p<0.01; *** p<0.001.

## Appendix S9 Residual diagnostics

**a.** Distribution of predicted random intercepts for 137 countries

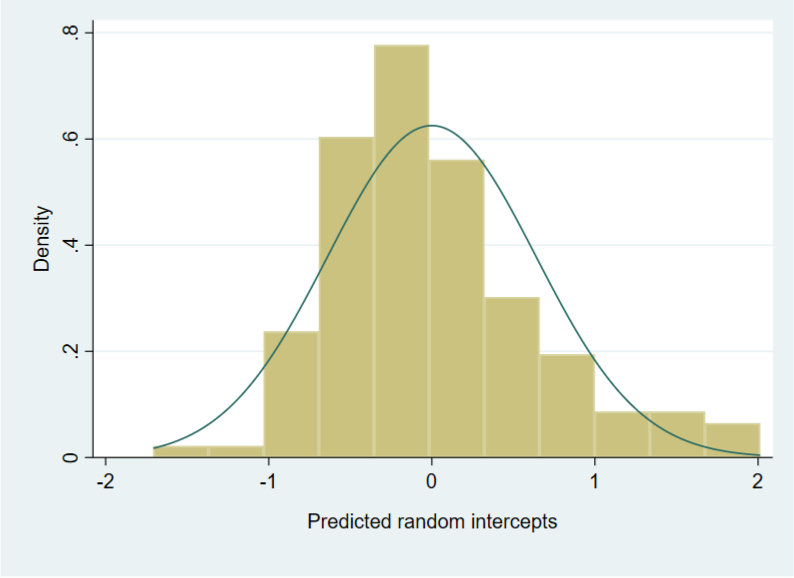


**b.** Distribution of predicted random slopes for 137 countries

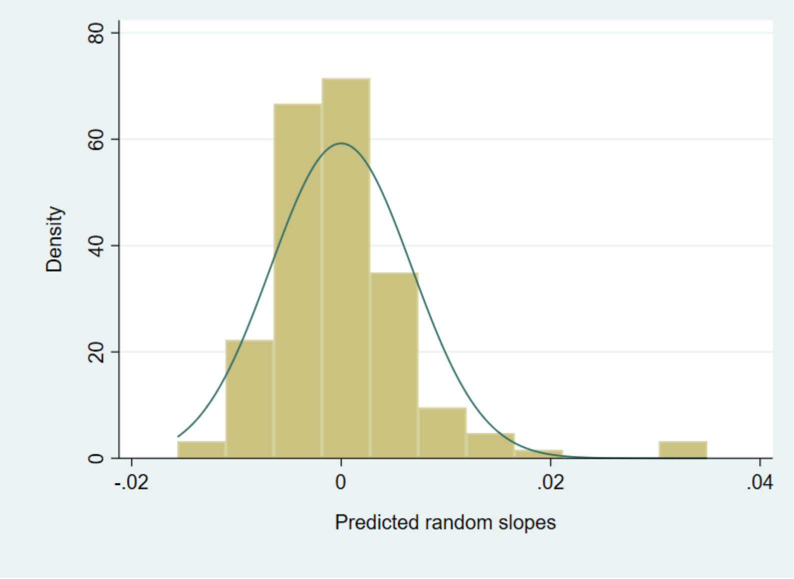


Figure S9. Residual diagnostics. This study used the empirical Bayes prediction method to assign values to country-specific random intercepts and random slopes, and assumed that they have a normal distribution. **a.** Distribution of predicted random intercepts for 137 countries. **b.** Distribution of predicted random slopes for 137 countries. Predicted random slopes are positively skewed, but in general predicted random effects are close to normal distributions.

## Appendix S10 Cross validation of the study results

To check the stability and robustness of the study results, a cross-validation method was applied. First, we randomly assigned 50% of the data for individual countries as the training data, and saved the remaining data for testing (validation). Second, we ran the regression (Equation (1) in the main text) using the training data to obtain coefficient estimates and country-specific random intercepts and slopes of time. Third, we predicted the case doubling time (DT) for the testing data using the estimated model and compared the fitted with observed DT (Table S10.1). The equation for prediction is given by Equation (S1) in Appendix S11. Finally, the DT regression was run on the testing data; the results were compared with those from the training data and from the full sample (Table S10.2).

Table S10.1 shows that the root mean squared error (RMSE) calculated was only marginally higher for testing (0.679) than for training (0.657) data. The correlation coefficient between fitted and observed DT was only slightly lower for the testing data (0.802) than for the training data (0.816). These results suggest that the study results seemed robust to different datasets. In addition, Table S10.2 shows that the coefficient estimates were consistent for regressions using the full sample (the main model), the training data and testing data.

We also performed random forest algorithm using Stata command *rforest* and compared the prediction accuracy with that of the random-effect growth-curve model. The RMSE converged prior to100 iterations; thus we set iterations to be 100. The lowest validation RMSE (calculated against the testing data) occurred at 0.22 when the number of randomly selected variables at each split was 7. This result shows that random forest model had a slightly higher prediction accuracy than the empirical model used in this study. For the present study, the goal was to estimate the relative effectiveness of a wide range of policy measures (which increased the RMSE); hence the random forest model was used for validation only.

Table S10.1 Prediction accuracy based on RMSE and correlation coefficient

| | Root mean squared error | Correlation coefficient between fitted and observed DT |
|---|---|---|
| Own prediction from training data (50% of the sample) | 0.657 | 0.816 |
| Prediction for testing data (50% of the sample) | 0.679 | 0.802 |

Table S10.2 Results from regressions using training and testing data

| Y: case doubling time (in log days) | **Main model** | **From training data** | **From testing data** |
|---|---|---|---|
| Predictors | Coef. | Coef. | Coef. |
| No. of days since the most recent implementation | | | |
| Partial school closures | 0.0138*** | 0.0141*** | 0.0135*** |
| Full school closures | 0.0040** | 0.0039** | 0.0040** |
| Partial workplace closures | 0.0009 | 0.0008 | 0.001 |
| Full workplace closures | 0.0141*** | 0.0135*** | 0.0146*** |
| Cancellation of public events | 0.0021 | 0.0021 | 0.0022 |
| Restriction on gatherings to 100 people | 0.0028 | 0.0034 | 0.002 |
| Restriction on gatherings to 10 people | 0.003 | 0.0029 | 0.003 |
| Stay-at-home requirements | 0.0015 | 0.0017 | 0.0014 |
| Bans on arrivals from some regions | -0.0004 | -0.0015 | 0.0008 |
| Bans on arrivals from all regions | 0.0018 | 0.0017 | 0.0017 |
| Coordinated public campaigns | 0.0039 | 0.0031 | 0.0043 |
| Widespread testing | -0.0023 | -0.0019 | -0.0026 |
| Comprehensive contact tracing | -0.0019 | -0.0019 | -0.0018 |
| Face covering required in some places | -0.0003 | 0.0002 | -0.0011 |
| Face covering required in all places | -0.0001 | -0.0002 | 0.0002 |
| COVID-19 vaccine rollout | 0.0196** | 0.0195** | 0.0203** |
| Government effectiveness scores (-25 – 25) | 0.0226* | 0.0229* | 0.0202* |
| Number of weeks from first death to the earliest policy | -0.014 | -0.0126 | -0.0116 |
| Population (log) | -0.1607*** | -0.1485*** | -0.1564*** |
| Population aged 65 and above (%) | 0.0344 | 0.0279 | 0.0289 |
| Number of people per $km^2$of land area | -0.0083* | -0.0066 | -0.0077* |
| Universal Health Coverage service coverage index (0–100) | 0.0046 | 0.0023 | 0.0033 |
| GDP per capita adjusted by purchasing power parity (log) | -0.2255* | -0.1998 | -0.1962 |
| Death caused by communicable diseases (%) | 0.0024 | 0.0032 | 0.0017 |
| Africa (=1) | 0.0468 | -0.0648 | 0.0686 |
| Asia (=1) | 0.2627 | 0.2155 | 0.2541 |
| North America (=1) | -0.3071 | -0.2842 | -0.2881 |
| South America (=1) | -0.3705 | -0.3754 | -0.3365 |
| Oceania (=1) | 0.5917* | 0.5 | 0.3409 |
| Number of countries | 137 | 137 | 137 |
| Number of observations (country days) | 42102 | 20959 | 21143 |

Note: All models included the time trend, quadratic terms of time and policy variables, and country-specific random intercepts and random coefficients of time. Country-clustered robust standard errors were used. Legend: * p<0.05; ** p<0.01; *** p<0.001.

## Appendix S11 Predicted versus observed trends of case doubling time for 137 countries

To examine the validity of the model, we predicted the case doubling time (DT) for individual countries over time, and compared country-specific fitted regression lines with the real data, as shown by figures below. Based on Equation (1) in the main text, the fitted DT was calculated as follows:

$$\hat{Y}_{it} = \widehat{\alpha_0} + \widehat{\alpha_1}T_{it} + \widehat{\alpha_2}T_{it}^2 + \sum_{j=1}^{6}(\widetilde{\widetilde{\boldsymbol{C}}}'_j\widetilde{\boldsymbol{\beta}}_j + \widetilde{\widetilde{\boldsymbol{D}}}'_j\widetilde{\boldsymbol{\gamma}}_j) + \sum_{k=1}^{5}(\widetilde{\widetilde{\boldsymbol{H}}}'_k\widetilde{\boldsymbol{\delta}}_k + \widetilde{\widetilde{\boldsymbol{G}}}'_k\widetilde{\boldsymbol{\theta}}_k) + \widetilde{\widetilde{\boldsymbol{X}}}'\widetilde{\boldsymbol{\zeta}} + \widehat{\eta_{1i}} + \widehat{\eta_{2i}}T_{it} \quad . \tag{S1}$$

Random intercepts and random slopes for individual countries were not estimated with other model parameters. Instead, we assigned them values by using empirical Bayes predictions. The standard practice is to assume that $\eta_{1i}$ and $\eta_{2i}$ have a normal prior distribution with a mean of zero and the variance previously estimated by the maximum likelihood method. The normality assumption was verified by residual diagnostics, as described in Appendix S9.

The following figures are displayed in the countries' alphabetic order. The scattered black dots represent observed data. The solid blue line is the predicted case doubling time (log). The shaded area is the 95% confidence interval for the fixed part of the model.

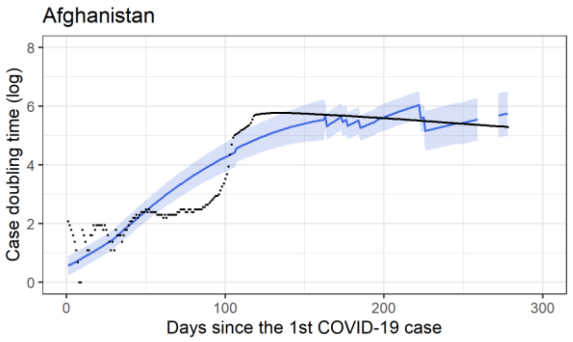


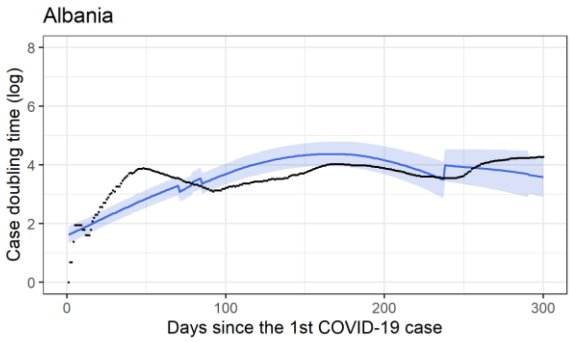


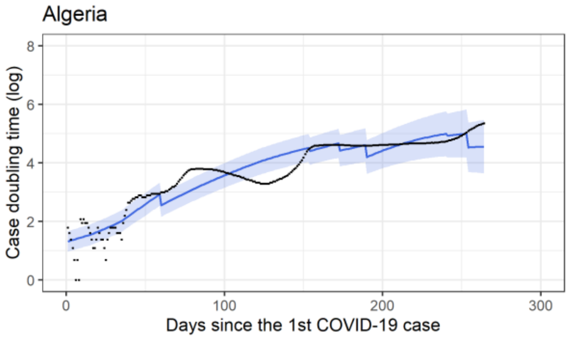


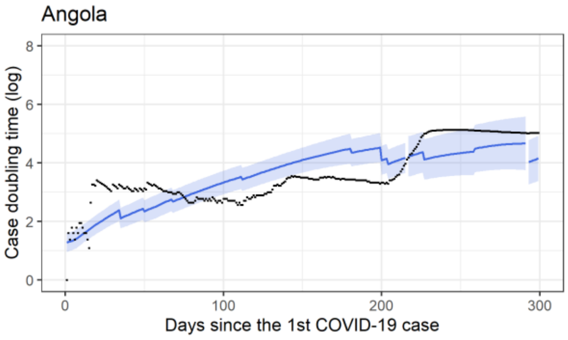

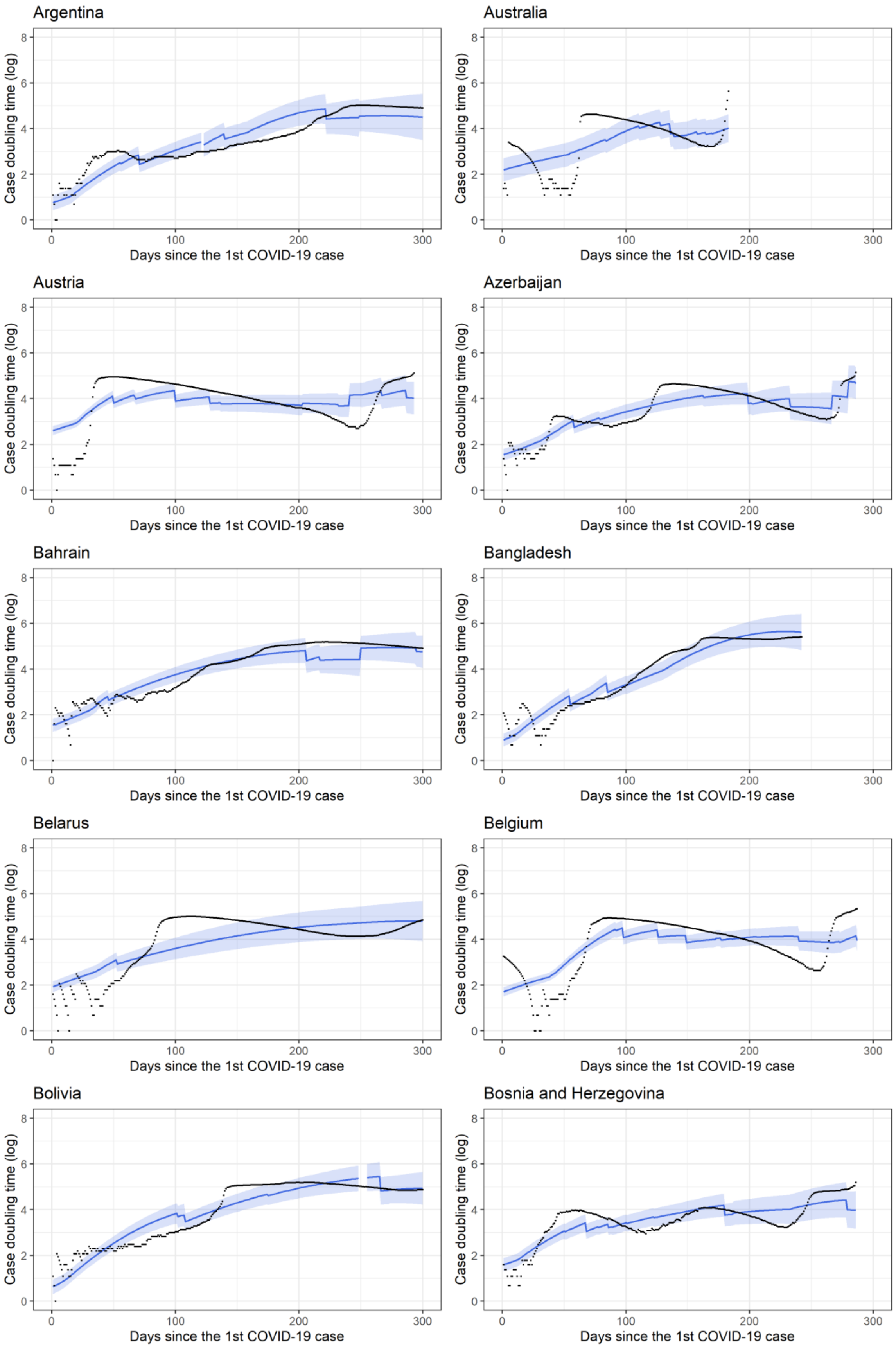
Argentina
Australia
Austria
Azerbaijan
Bahrain
Bangladesh
Belarus
Belgium
Bolivia
Bosnia and Herzegovina
Case doubling time (log)
Days since the 1st COVID-19 case
0
2
4
6
8
0
100
200
300

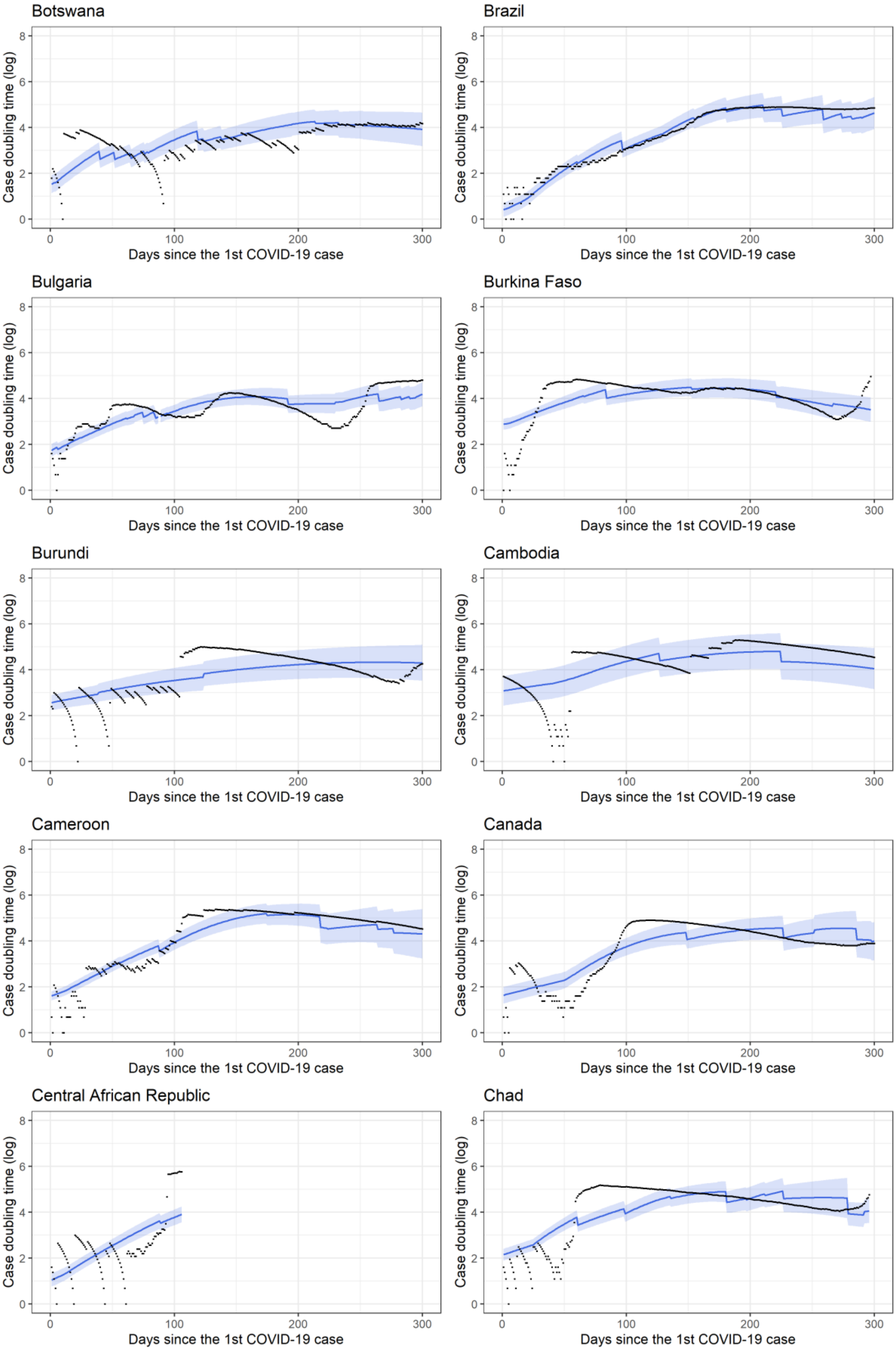
Botswana
Brazil
Bulgaria
Burkina Faso
Burundi
Cambodia
Cameroon
Canada
Central African Republic
Chad
Case doubling time (log)
Days since the 1st COVID-19 case
0
2
4
6
8
0
100
200
300

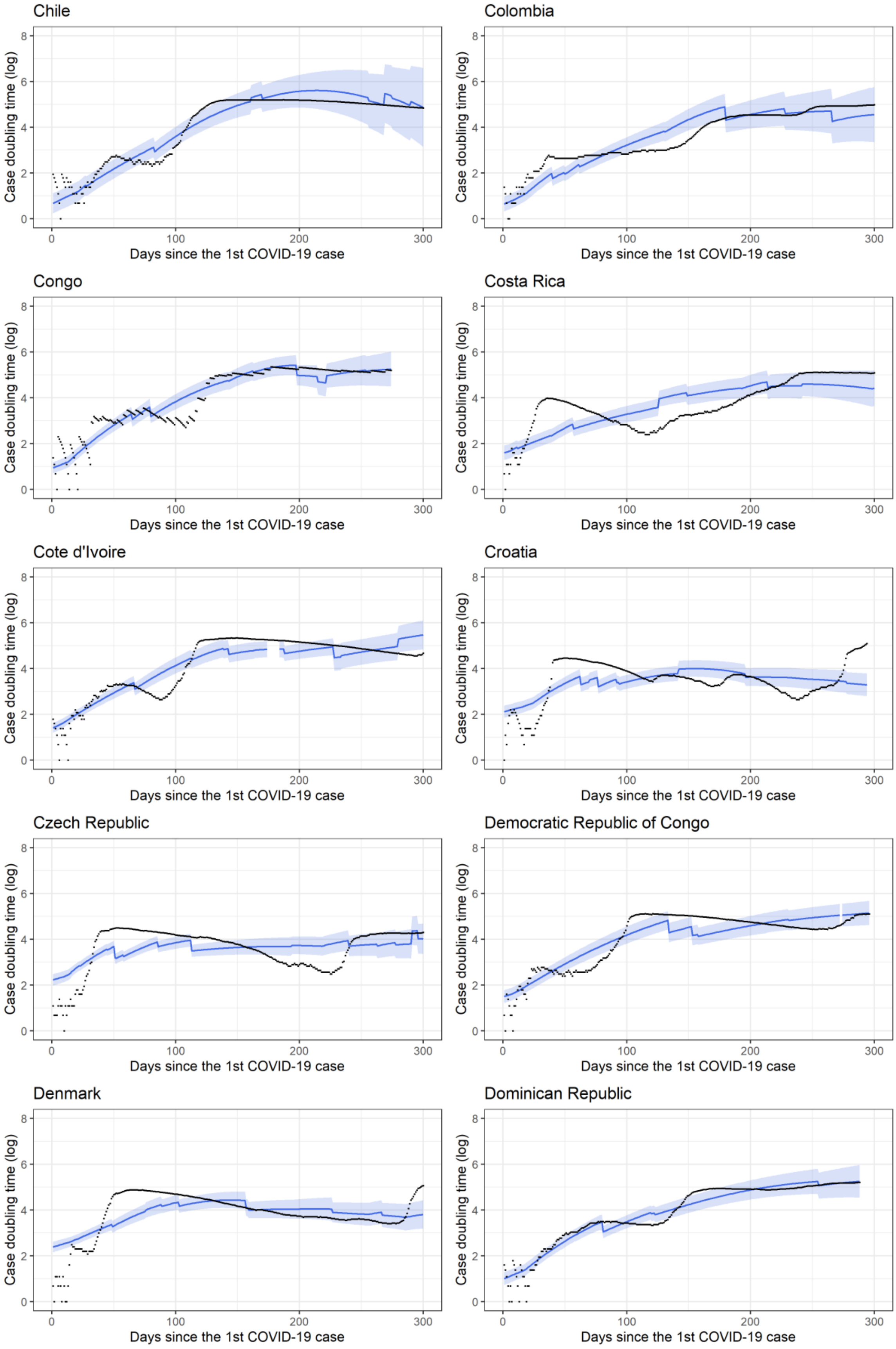
Chile
Colombia
Congo
Costa Rica
Cote d'Ivoire
Croatia
Czech Republic
Democratic Republic of Congo
Denmark
Dominican Republic
Case doubling time (log)
Days since the 1st COVID-19 case
0
2
4
6
8
0
100
200
300

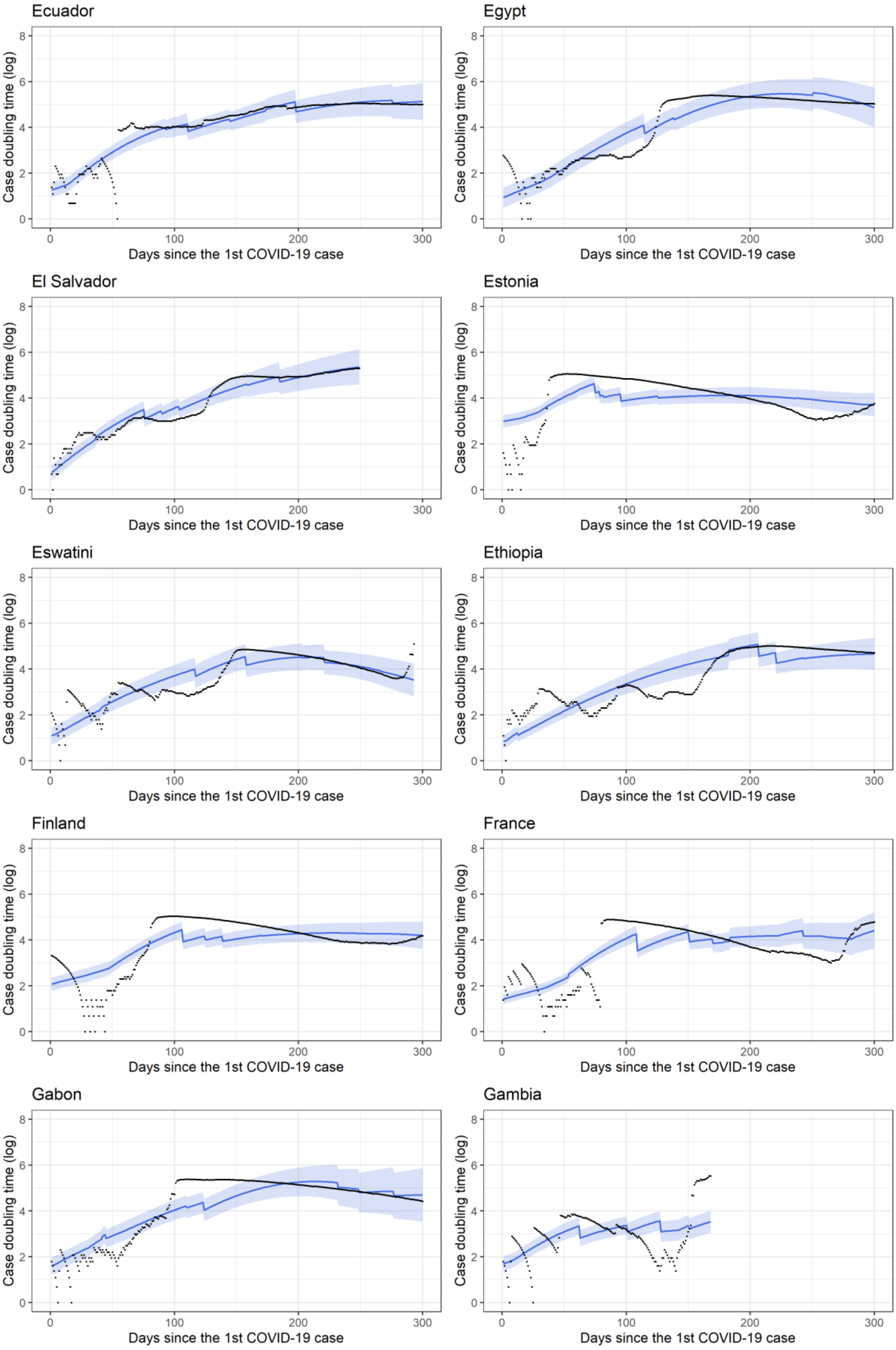
Ecuador
Egypt
El Salvador
Estonia
Eswatini
Ethiopia
Finland
France
Gabon
Gambia
Case doubling time (log)
0
2
4
6
8
Days since the 1st COVID-19 case
0
100
200
300

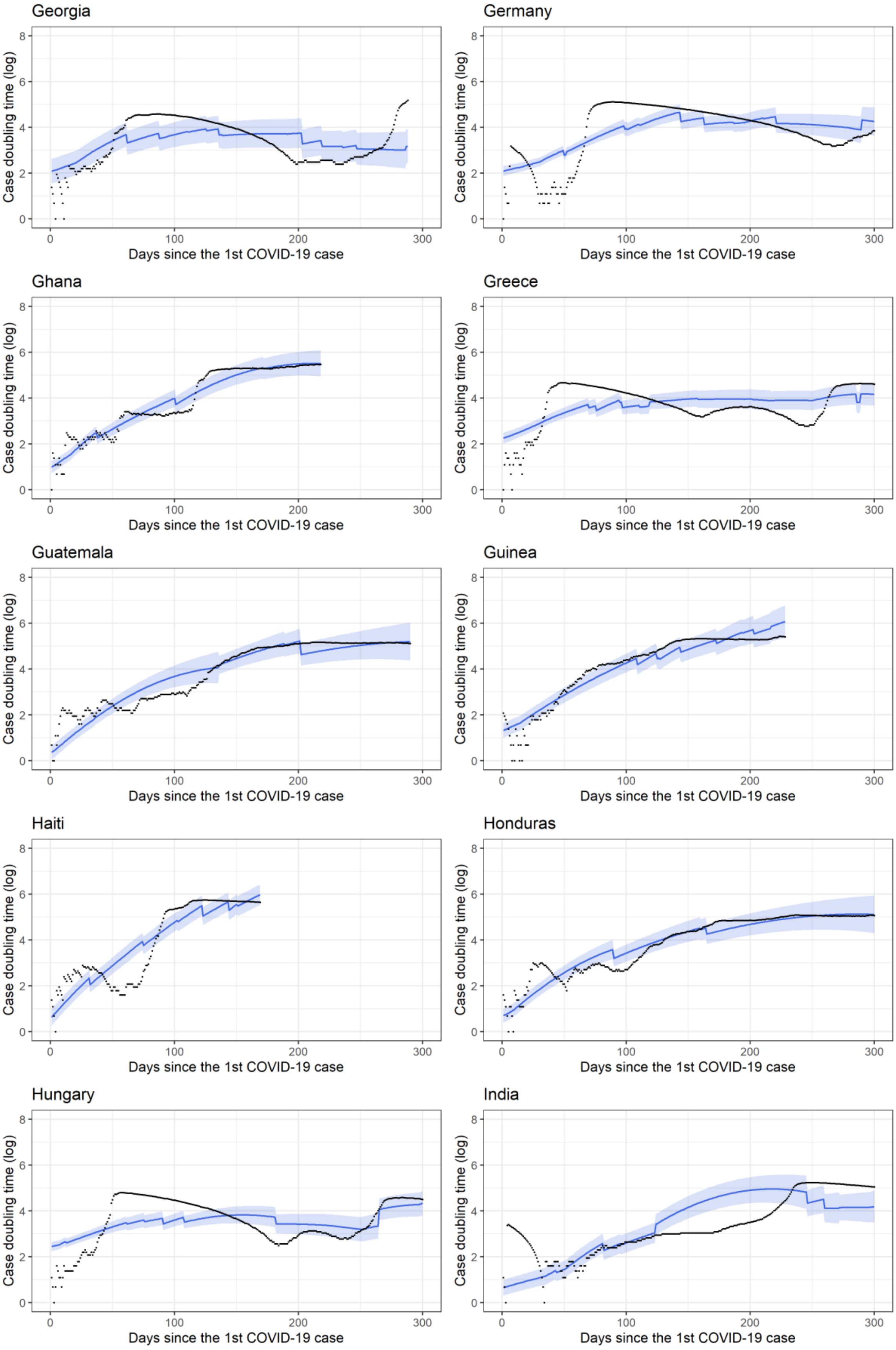
Georgia
Germany
Ghana
Greece
Guatemala
Guinea
Haiti
Honduras
Hungary
India
Case doubling time (log)
0
2
4
6
8
Days since the 1st COVID-19 case
0
100
200
300

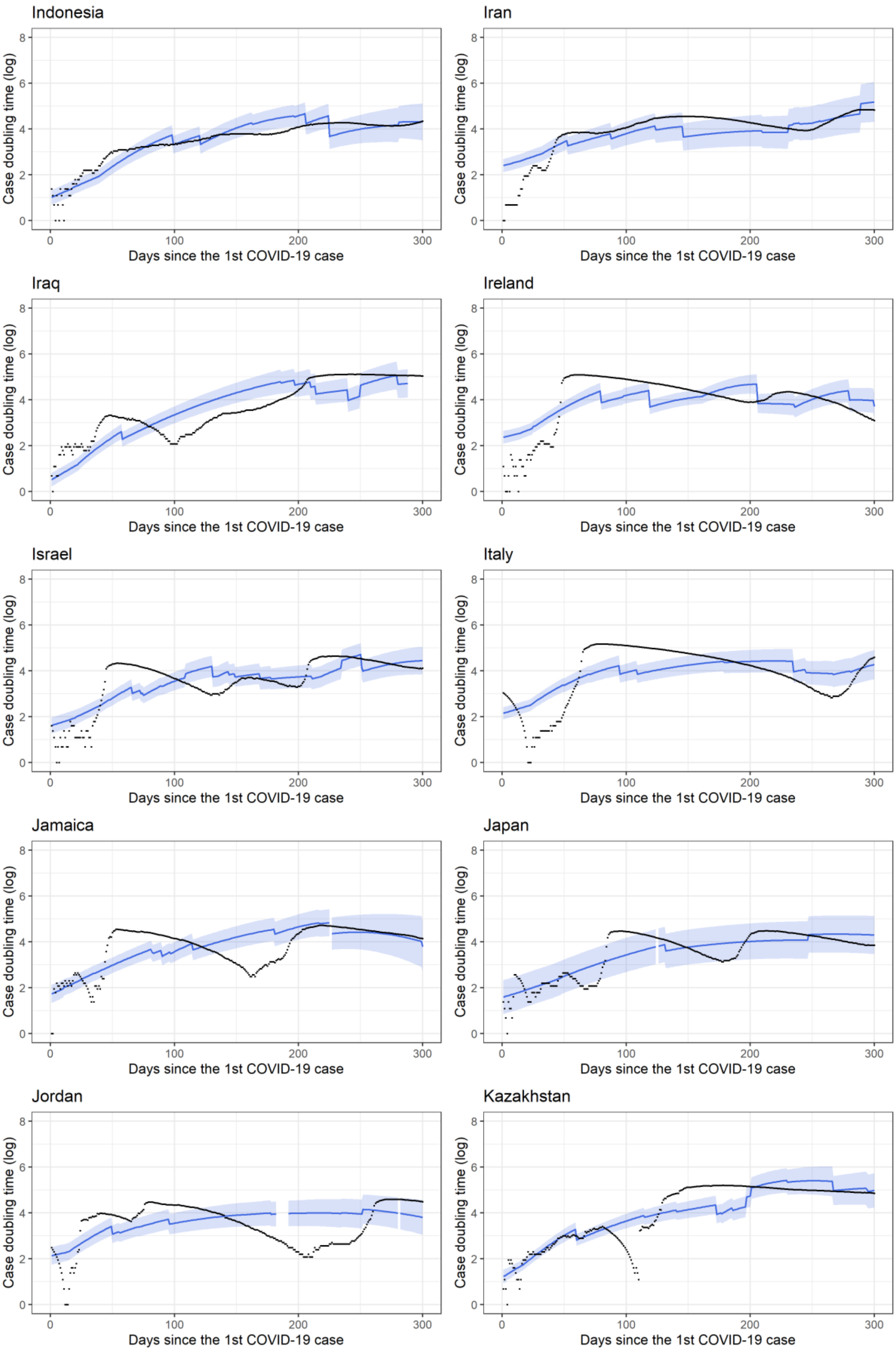
Indonesia
Iran
Iraq
Ireland
Israel
Italy
Jamaica
Japan
Jordan
Kazakhstan
Case doubling time (log)
Days since the 1st COVID-19 case
0
2
4
6
8
0
100
200
300

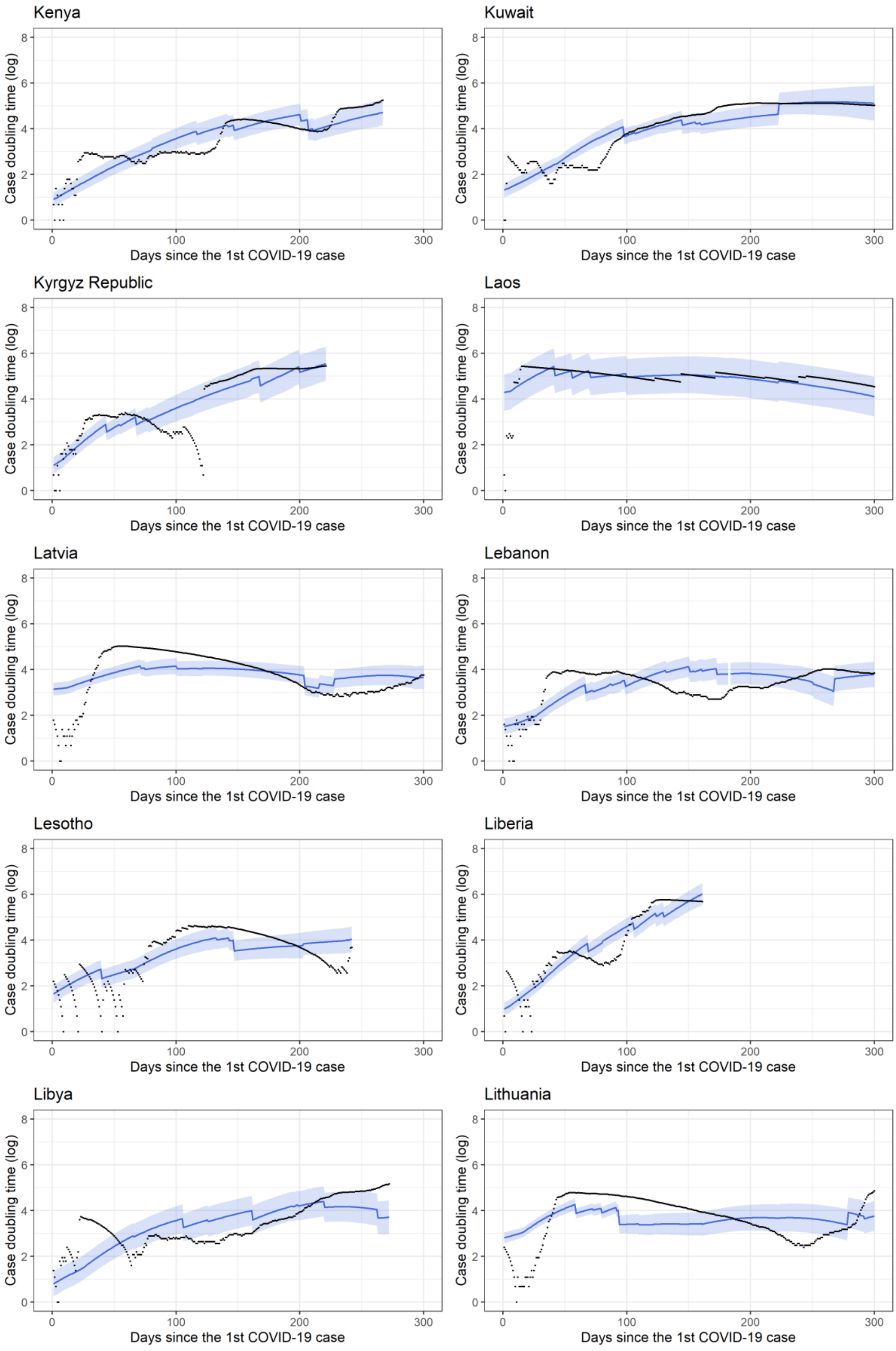
Kenya
Kuwait
Kyrgyz Republic
Laos
Latvia
Lebanon
Lesotho
Liberia
Libya
Lithuania
Case doubling time (log)
Days since the 1st COVID-19 case
0
2
4
6
8
0
100
200
300

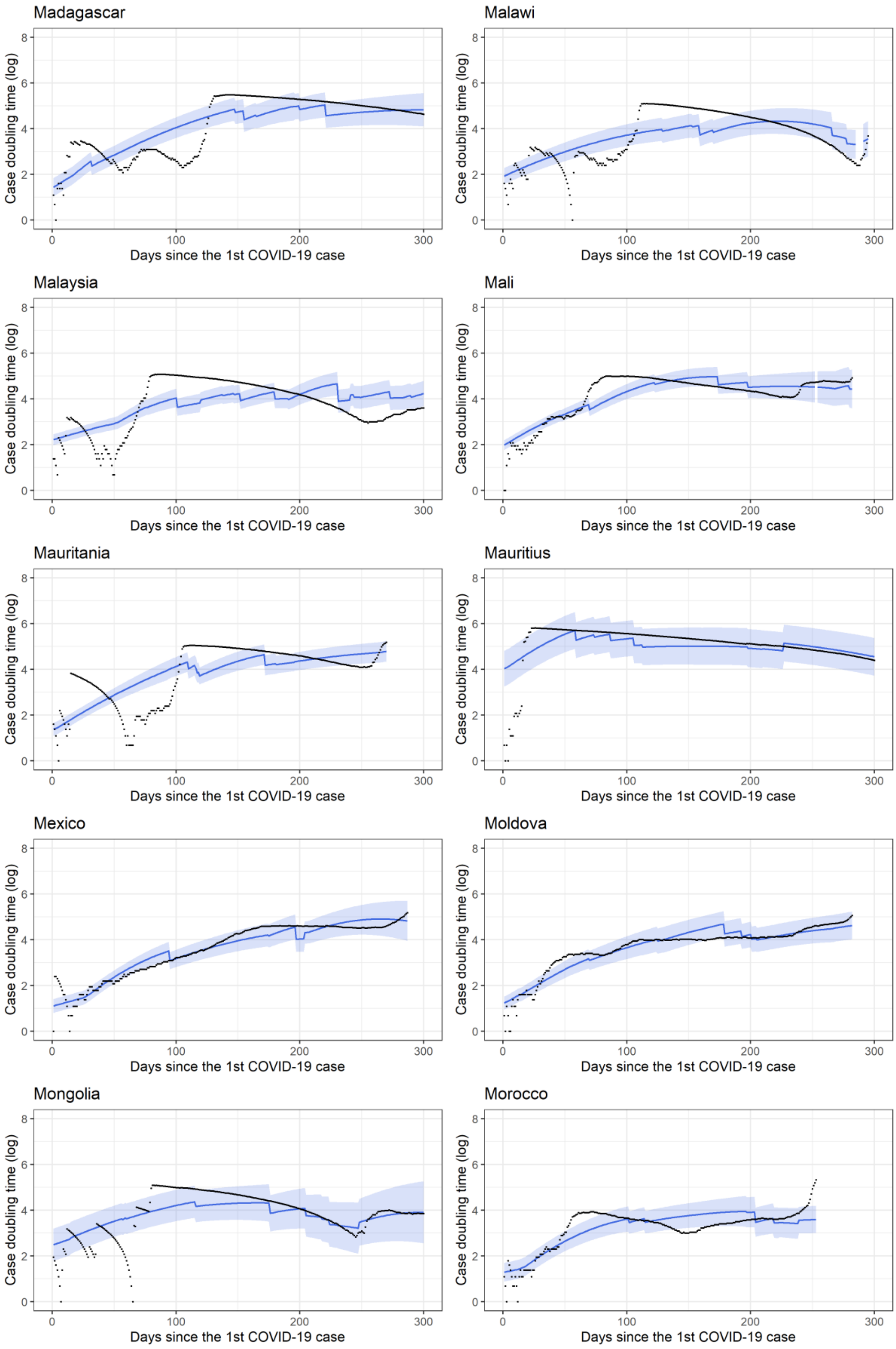
Madagascar
Malawi
Malaysia
Mali
Mauritania
Mauritius
Mexico
Moldova
Mongolia
Morocco
Case doubling time (log)
Days since the 1st COVID-19 case
0
2
4
6
8
0
100
200
300

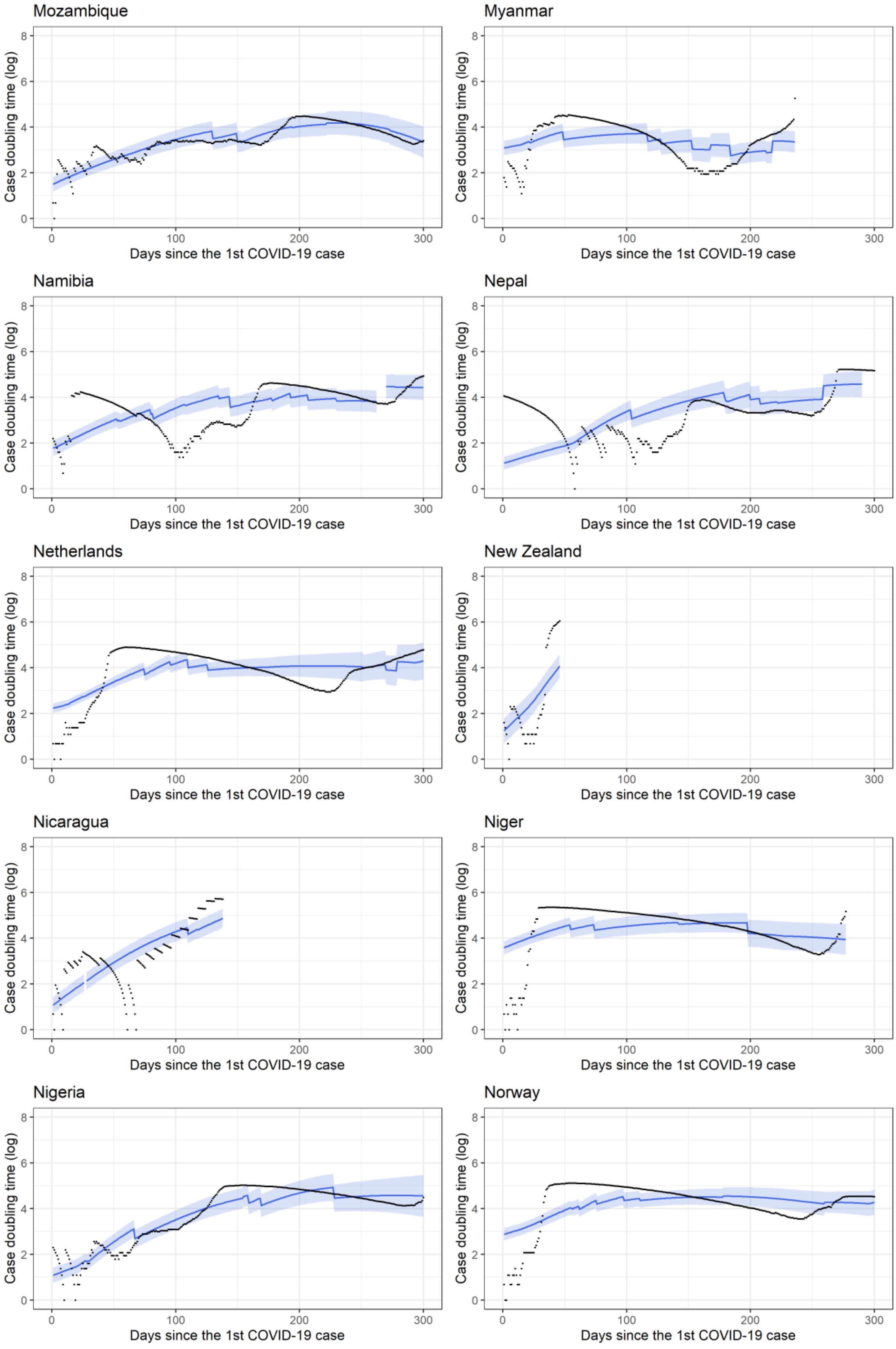
Mozambique
Myanmar
Namibia
Nepal
Netherlands
New Zealand
Nicaragua
Niger
Nigeria
Norway
Case doubling time (log)
Days since the 1st COVID-19 case
0
2
4
6
8
0
100
200
300

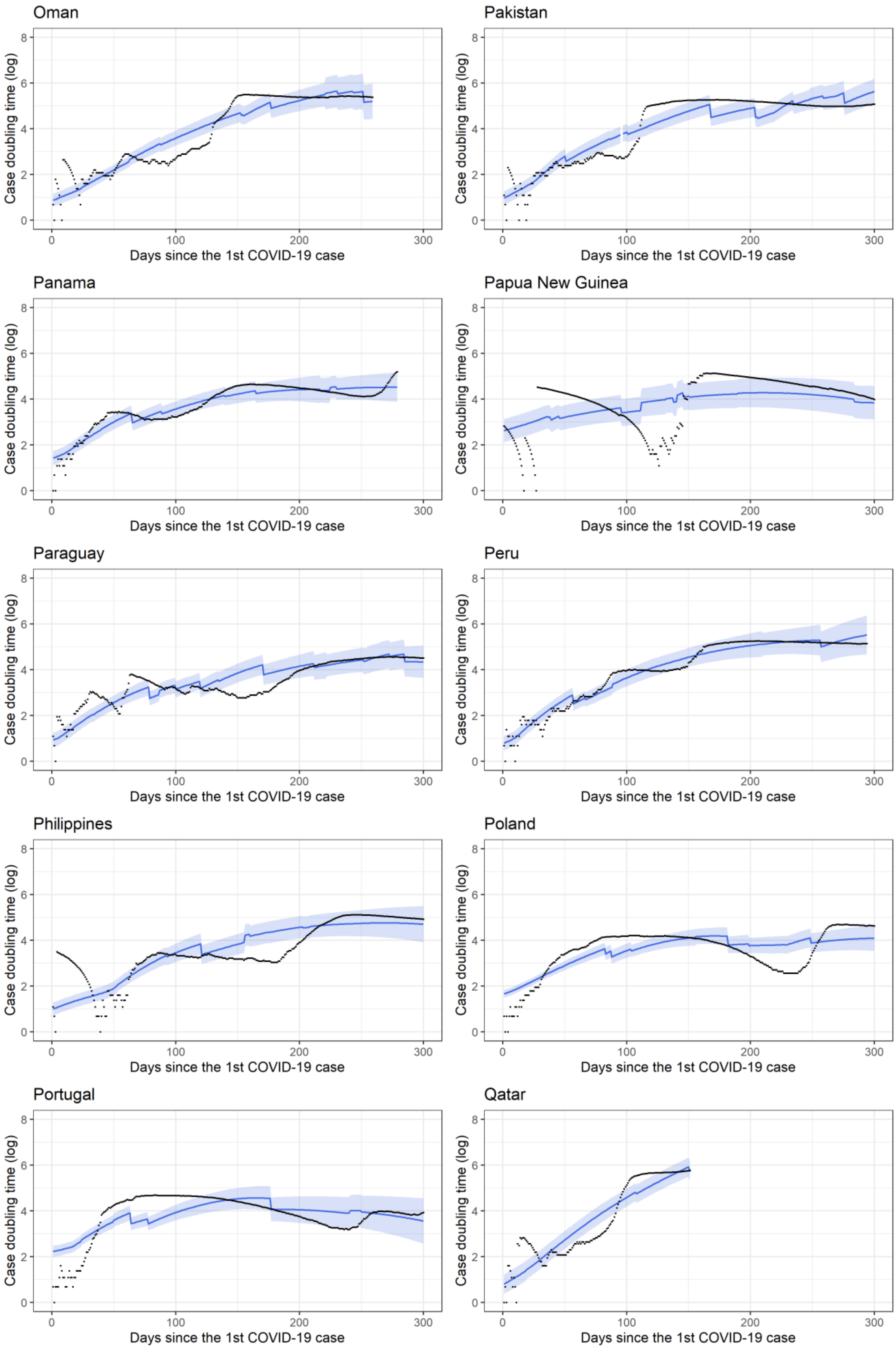
Oman
Pakistan
Panama
Papua New Guinea
Paraguay
Peru
Philippines
Poland
Portugal
Qatar
Case doubling time (log)
Days since the 1st COVID-19 case
0
2
4
6
8
0
100
200
300

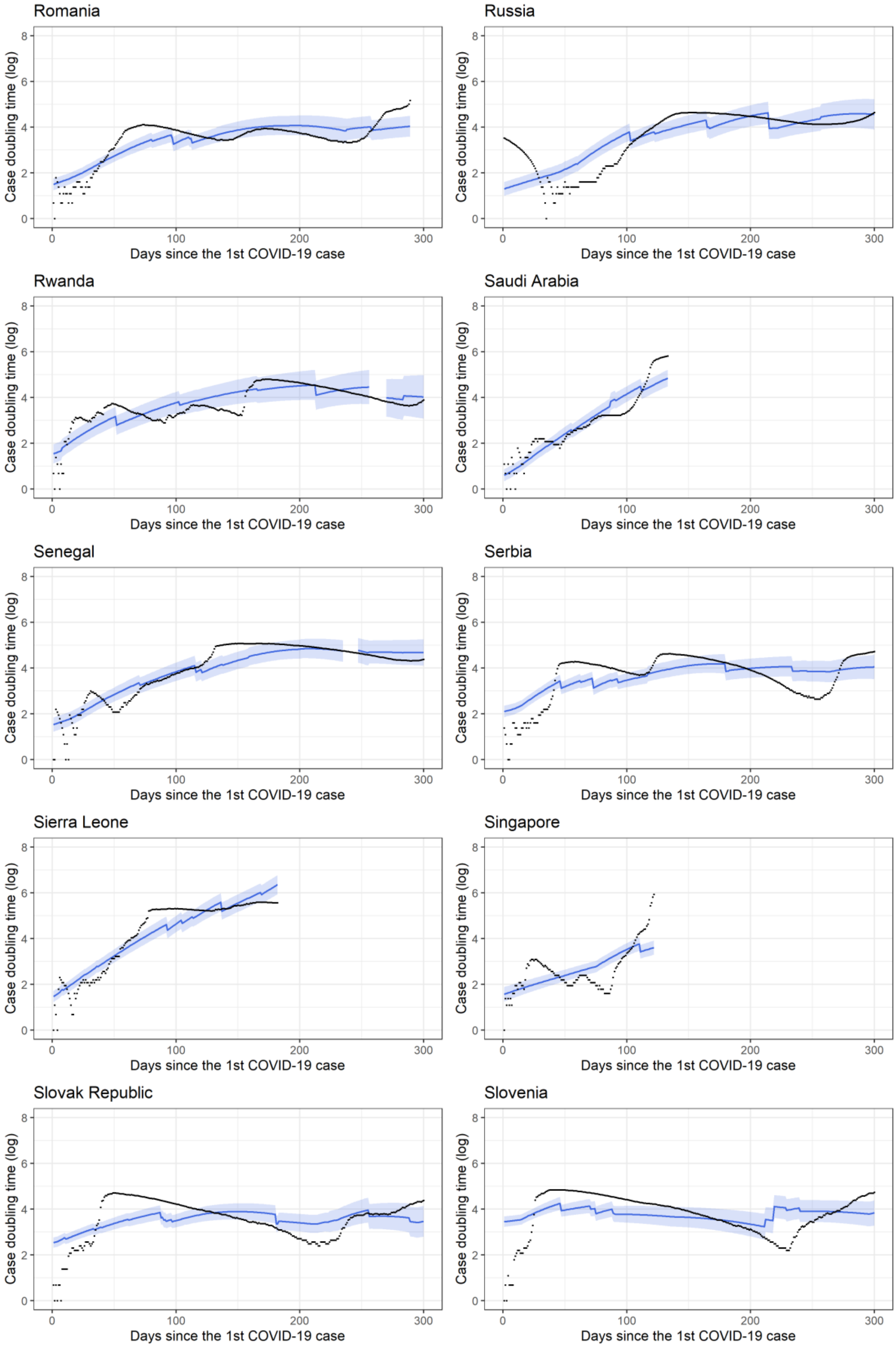

Romania
Russia
Rwanda
Saudi Arabia
Senegal
Serbia
Sierra Leone
Singapore
Slovak Republic
Slovenia
Case doubling time (log)
Days since the 1st COVID-19 case
0
2
4
6
8
0
100
200
300

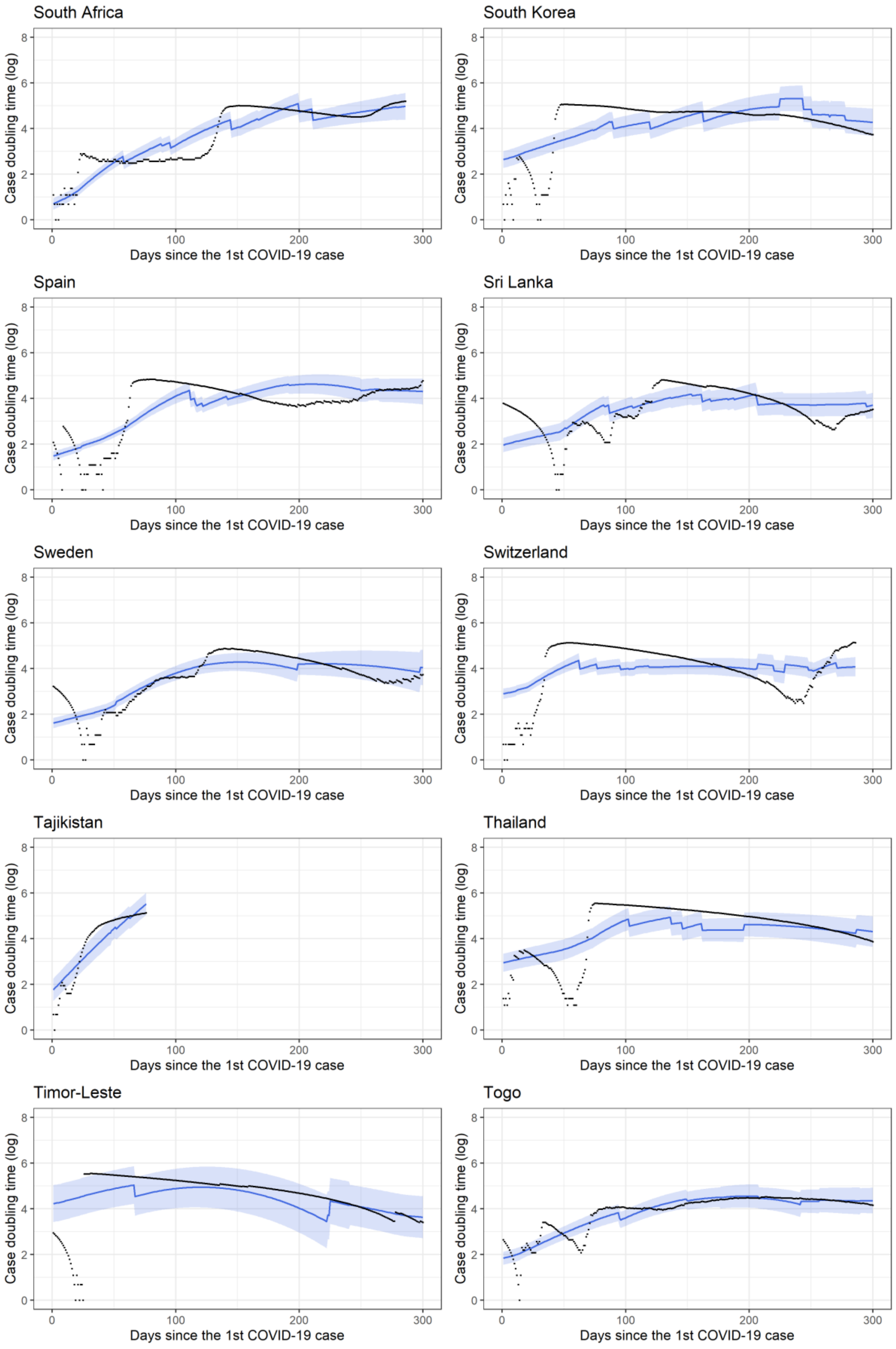
South Africa
South Korea
Spain
Sri Lanka
Sweden
Switzerland
Tajikistan
Thailand
Timor-Leste
Togo
Case doubling time (log)
Days since the 1st COVID-19 case
0
2
4
6
8
100
200
300

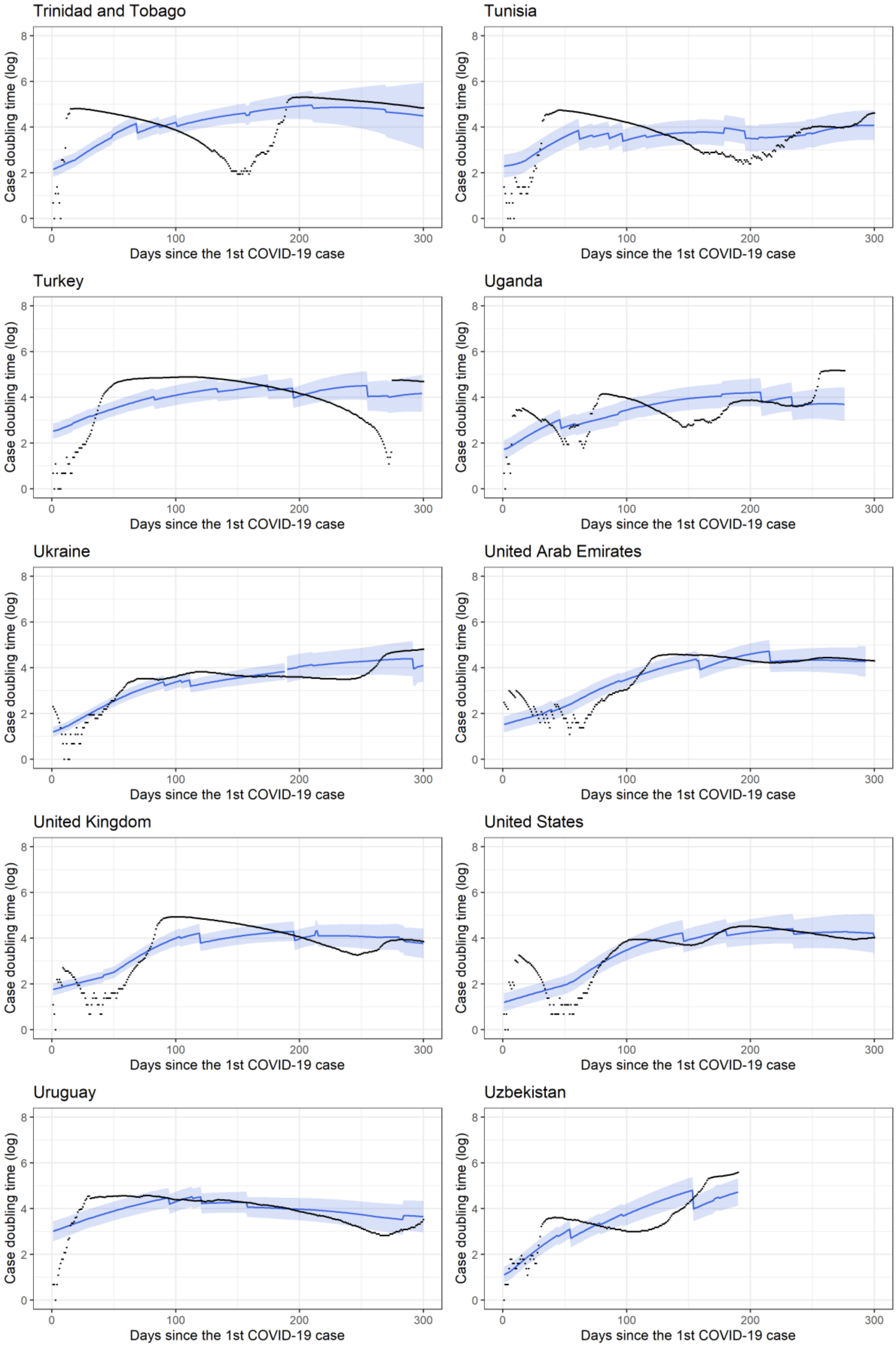
Trinidad and Tobago
Tunisia
Turkey
Uganda
Ukraine
United Arab Emirates
United Kingdom
United States
Uruguay
Uzbekistan
Case doubling time (log)
Days since the 1st COVID-19 case
0
2
4
6
8
100
200
300

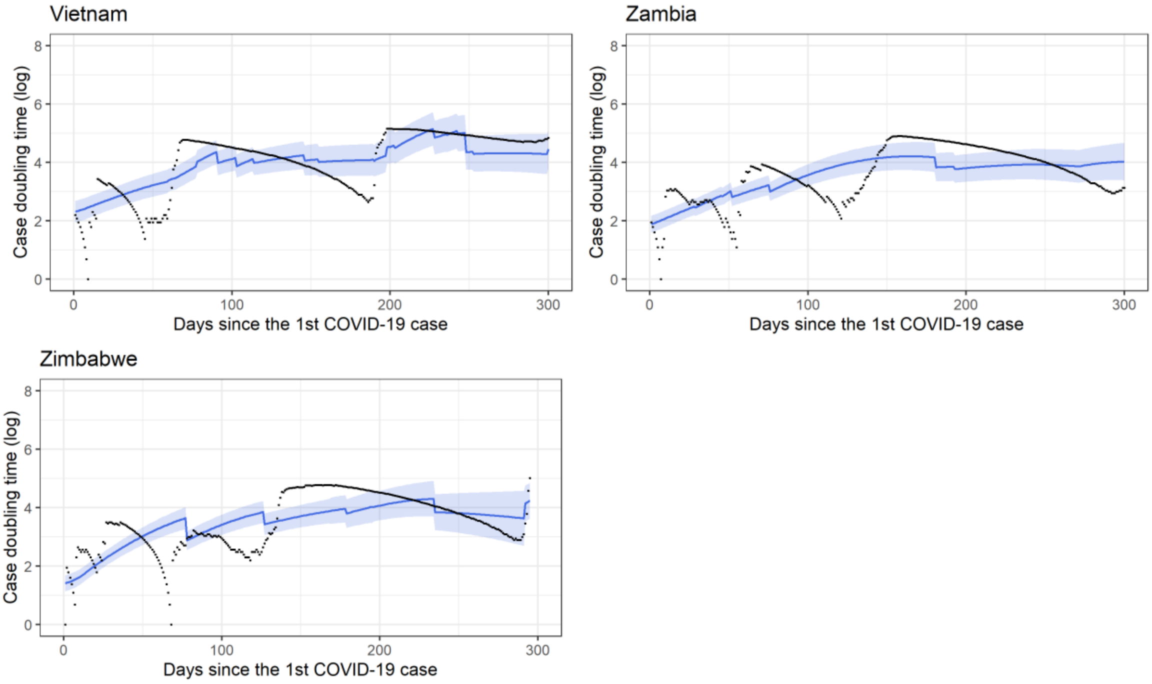


Figure S11 Predicted versus observed trends of case doubling time for 137 countries
